# Study protocol for a triple-blind randomised controlled trial evaluating a machine learning-based predictive clinical decision support tool for internet-delivered cognitive behaviour therapy (ICBT) for depression and anxiety^☆^

**DOI:** 10.1016/j.invent.2025.100816

**Published:** 2025-03-03

**Authors:** Pontus Bjurner, Nils Hentati Isacsson, Fehmi Ben Abdesslem, Magnus Boman, Erik Forsell, Viktor Kaldo

**Affiliations:** a Centre for Psychiatry Research, Department of Clinical Neuroscience, Karolinska Institutet, Stockholm Health Care Services, Region Stockholm, Sweden; b Department of Computer Science, RISE Research Institutes of Sweden, Stockholm, Sweden; c Division of Psychiatry, University College London, London, England, United Kingdom; d Department of Medicine Solna, Division of Clinical Epidemiology, Karolinska Institutet, Stockholm, Sweden; e Department of Psychology, Faculty of Health and Life Sciences, Linnaeus University, Växjö, Sweden

## Abstract

**Introduction:**

Therapist-supported internet-based Cognitive Behavioural Therapy (ICBT) has strong scientific support, but all patients are not helped, and further improvements are needed. Personalized medicine could enhance ICBT. One promising approach uses a Machine learning (ML) based predictive decision support tool (DST) to help therapists identify patients at risk of treatment failure and adjust their treatments accordingly. ICBT is a suitable clinical context for developing and testing such predictive DST's, since its delivery is quite flexible and can quickly be adapted for probable non-responders, for example by increasing the level and nature of therapist support, to avoid treatment failures and improve overall outcomes. This type of strategy has never been tested in a triple-blind randomised controlled trial (RCT) and has rarely been studied in ICBT.

The aim of this protocol is to expand on previous registered protocols with more detailed descriptions of methods and analyses before analyses is being conducted.

**Methods and analysis:**

A triple blind RCT comparing ICBT with a DST (DST condition), to ICBT as usual (TAU condition). The primary objective is to evaluate if the DST condition is superior to the TAU condition in decreasing diagnose-specific symptoms among patients identified to be at risk of failure. Secondary objectives are to evaluate if the DST improves functioning, interaction, adherence, patient satisfaction, and therapist time efficiency and decreases the number of failed treatments. Additionally, we will investigate the therapists' experience of using the DST.

Patients and therapists have been recruited nationally. They were randomised and given a sham rationale for the trial to ensure allocation blindness. The total number of patients included was 401, and assessments were administered pre-treatment, weekly during treatment, at post-treatment and at 12-month follow-up. Primary outcome is one of the three diagnosis-specific symptom rating scales for respective treatment and primary analysis is difference in change from pre- to post-treatment for at-risk patients on these scales.

**Human ethics and consent to participate:**

Informed consent to participate in the study was obtained from all participants. Both therapists and patients are participants in this trial. For patients, informed consent to participate in the study was obtained when they registered interest for the study via the study's secure web platform and carried out initial screening before the diagnostic and fit for treatment assessment, they first received the research subject information and were asked for consent by digitally signing that they had read and understood the information. For therapists who were part of the study, consent was requested after they had registered their interest. Therapists then received an email with a link to the study's secure web platform with the research person's information and were asked for consent by digitally signing that they had read and understood the information. All documents are stored in secure, locked filing cabinets on the clinic's premises or on a secure digital consent database.

**Approval committee:**

Approved by the Swedish Ethical Review Authority (SERA), record number 2020–05772.

## Highlights

• First study evaluating a fully automatic machine learning-based predictive clinical decision support tool for ICBT.
• First study to use a triple blind randomised controlled trial to evaluate the clinical usefulness of an Adaptive Treatment Strategy.
• Large number of participants and therapists.

## 1 Introduction

### 1.1 Background and rationale

As reported in the latest World Health Organization (WHO) World Mental Health Report, a large proportion of the global population suffer from mental disorders, with depression and anxiety being the largest groups (Organization WH, 2022). This leads to individual distress and negative societal consequences showing the need for cost-effective and efficient treatments. Cognitive Behavioural Therapy (CBT) is currently the most evidence-based form of psychotherapy (Hofmann et al., 2012). Likewise, internet-based Cognitive Behavioural Therapy (ICBT), especially with therapist guidance, has accumulated strong scientific support during the last two decades (Andersson et al., 2019a; Karyotaki et al., 2021). The evidence for therapist guided ICBT shows that it can be as effective as face-to-face therapy (Andersson et al., 2019a; Carlbring et al., 2018; McCrone et al., 2004). Despite the success of psychological interventions such as CBT and ICBT the fact remains that not all patients are helped and some even get worse after treatment (Andersson et al., 2019b; Rozental et al., 2017). It has been estimated that 30–60 % show no significant improvements after treatment (Lambert and Ogles, 2004) and that 5–10 % have deteriorated (Rozental et al., 2014; Slade et al., 2008). Consequently, there is a need to improve the effects of psychological treatments.

Personalized medicine aims to identify which patient would benefit from which type of care and to adapt the care process to the individual's needs (Auffray and Hood, 2012). This model could be used to enhance outcomes of psychological treatments. Using baseline data to match a patient to a treatment, has thus far failed to show coherent results (Knopp et al., 2013; Schneider et al., 2015). Innovative variants, such as models where more specific CBT components are combined to fit an individual patient's profile assessed at baseline have been suggested (Furukawa et al., 2021), but overall, there is not enough evidence that we can make prospective matching predictions accurate enough to use in clinical practice, so we also need to work with other models within the field of Personalized Medicine.

Predictions of a patient's outcome based on data collected during treatment can be highly useful. Using early weekly symptom ratings in ICBT has been shown to be successful in predicting treatment failures (Forsell et al., 2020). This can inform the therapist and/or the patient whether it is worth continuing with the intervention or to adjust it in some way to better fit the patient's needs and current situation. It has further been found that therapists are not very good at predicting outcome for their patients, especially not negative outcomes (Forsell, 2020; Hannan et al., 2005). In general, therapists seem to be overly optimistic in their judgments of patient's progress.

Continuous monitoring of symptoms has been used in several studies to detect patients at risk of being a non-responder in a more reliable way than psychotherapist's own predictions (Lambert, 2015) and has led to fewer failed treatments (Shimokawa et al., 2010). Thus, to help therapists identify which patients are at risk of failure and to guide them in how to adapt the treatment seems to be a promising approach. This strategy has rather successfully been applied to psychological treatments and has been labelled Routine Outcome Monitoring (ROM) (Lambert et al., 2001), Patient Focused Feedback (Lutz et al., 2015), Measurement-based care (Lewis et al., 2018), or Adaptive Treatment Strategies (ATS) (Forsell et al., 2019). A growing number of studies have shown promising results when therapists are supported in identifying and adapting treatments to patients at risk of failure, and two recent meta-analyses have found small to medium positive effects (Rognstad et al., 2022; de Jong et al., 2021). These effects seem to be further enhanced when the identification of at-risk patients is complemented with decision support tools (DST) and providing guidance to therapists on how to adapt treatment (de Jong et al., 2021; Lambert et al., 2018).

In a previous randomised clinical trial, a DST was used to identify patients in ICBT for insomnia at risk of treatment failure, which increased treatment effects for those by providing an ATS (Forsell et al., 2019). However, the semi-manual classification routine consumed valuable therapist time and its predictive power leaves room for improvement. Machine learning (ML) methods could be a solution to these issues and are already showing promise as an accurate strategy for predicting outcomes in psychological treatments (Boman et al., 2019; Chekroud et al., 2021; Kaldo et al., 2021; Hentati Isacsson et al., 2024). ML algorithms can use a wide range of data sources to learn from a large set of examples (patients) and apply this knowledge on a new patient to, for example, predict final outcome (Hentati Isacsson et al., 2024; Schibbye et al., 2014). ML methods can outperform traditional prognostic methods, but so far, the actual usefulness in a clinical setting has not been tested in an RCT.

ICBT for specific psychiatric conditions is likely a highly suitable context for developing and testing ML-based predictions, DSTs, and an ATS. In ICBT a large amount of data is collected and stored, it is a highly standardized treatment format and thus probably more predictable. The focus of ICBT on a rather strictly selected patient group and the use of diagnose specific outcome measures, which have been shown to be more predictive compared to general outcome measures (Schibbye et al., 2014) is also a promising factor. It is also uncomplicated to integrate a DST, including the predictions, within an already digital treatment platform in a user-friendly way. Also, ICBT provides good opportunities to add new treatment modules and to increase the treatment intensity since it starts from a low level of about 10–15 min of therapist time each week, which can be quickly increased by intensifying therapist support and/or provide it via telephone, video, or face-to-face visits.

In an ongoing collaboration between the Internet Psychiatry Clinic (Titov et al., 2018), Karolinska institutet (KI), and KTH Royal Institute of Technology, the authors' research group have used over 6000 historical patients treated with ICBT for depression, social anxiety or panic disorder to train a ML model on data from for example repeated symptom measures, messages between therapist and patient, homework reports, and baseline factors. This ML model was used to predict treatment success (either 50 % reduction or under clinical cut-off) for each primary symptom outcome (Kaldo et al., 2021).

## 2 Evidence gap

Even though there is growing evidence that ROM or ATS using a predictive DST can increase the effects of psychological treatments, to our knowledge no RCT where therapists, patients, and assessors all have been blind to trial allocation exists. Also, almost all previous trials have been on ROM in traditional face-to-face treatments. Only one study has been in the context of internet delivered treatment, and this was for patients with Insomnia and its positive results might thus not generalize to other conditions (Forsell et al., 2019).

### 2.1 Aims, objectives, and hypotheses

The aim of this protocol is to expand on previous registered protocols with more detailed descriptions of methods and analyses before analyses is being conducted. The overall aim of the study itself is to test the clinical benefits of a ML-based predictive DST for ICBT and to evaluate how the DST affects therapists and their patient's receiving treatment for depression, social anxiety, or panic disorder during 12 weeks of ICBT.

More specifically the study objective is to evaluate if therapist supported ICBT, where therapists are guided by a DST in addition to the regular therapist manual (the DST condition) is superior to ICBT using only the regular therapist manual (the treatment as usual (TAU) condition). The authors hypothesize that the DST condition, in comparison to the TAU condition, will:

• Decrease the diagnosis specific symptoms (primary outcome) more during the treatment period, among patients identified to be at risk of failure (primary analysis).
  Related to this, secondary hypotheses are that the effect is more pronounced when patients identified as at-risk with only two weeks left in treatment are excluded, as well as when only patients identified with the highest level of risk (Dark Red, as defined below) are included.
• Decrease the proportion of failed treatments (neither a responder or remitter) among at-risk patients.
• Improve everyday functioning, health related quality of life, patient satisfaction, number of Adverse Events experienced by the patient, and need for further treatment for at-risk patients.
• Increase the therapists' amount of interaction with at-risk patients and time spent on at-risk patients and decrease it for not-at-risk patients.
• Increase the adherence to treatment among at-risk patients.
• Improve levels of symptoms, functioning, interaction, adherence, and other outcomes when all patients (also those not-at-risk) are included.
• Make therapists overall more time efficient, defined as the ratio of ‘decrease in symptoms / therapist time spent per patient’.
• Lead to therapists in the DST condition perceiving the DST in combination with the therapist manual as more helpful and credible than how the therapists in the TAU condition will perceive the therapist manual only.

We also hypothesize that for at-risk patients in the DST condition, the decrease of symptoms will be faster during the period from the time-point they are identified as at-risk and to the post-treatment measure, compared to their period from pre-treatment to the identification point and compared to the rate of symptom decrease from pre to post for all identified at-risk patients in the TAU condition.

Another hypothesis is that there will be a trend showing that the effect (on symptoms) for the DST condition, compared to the TAU condition, will be largest for at-risk patients detected earlier in treatment and then decrease the later in treatment the detection occurs, since there is less time for treatment adaptions to have effect due to the ICBT-programs being restricted to 12 weeks.

We also expect that in the DST condition, at-risk patients will decrease their symptoms as much as patients not-at-risk but to a lesser extent be defined as remitters after treatment, while in the TAU condition the at-risk patients will improve significantly less on both these measures than patients not-at-risk.

Differences in all outcome variables will be explored for patients not-at-risk, to examine indications of negative effects of being in the DST condition for this group.

The study will also in an exploratory way investigates therapists' overall experience of supervision, clinical routines, and guidance of their clinical decisions and if this differs between the two conditions. For therapists in the DST condition the study investigates how they experience applying treatment adaptations. Data from the TAU condition will be used to evaluate the balanced accuracy of the predictive ML model used in the DST, to compare its performance on this new sample of patients compared to the historical groups of patients it was trained on. Explorations of new predictive models and predictors will be made. Also, explorations of if the DST effect differs between ICBT-programs (i.e. for depression, social anxiety disorder, or panic disorder), and between patients of different levels of problem severity at intake.

The study also investigates what adaptations therapists use for at-risk patients in the DST condition and if they differ from at-risk patients in the TAU condition and from not-at-risk patients. The study further evaluates effects due to therapists or type of ICBT-program (i.e. for depression, social anxiety disorder, or panic disorder) on outcome, and if there is a training and experience effect where therapists' early patients benefit less than later patients.

In line with the views put forward by Klonsky (Klonsky, 2024) and a reply to this article by Vize and colleagues (Vize et al., 2024), the general aim of publishing this study protocol is to enhance transparency in the research process when investigating the above-described hypotheses. We acknowledge that a pre-registered protocol does not affect the risk for multiple-comparisons-related chance findings, but it makes it easy for future reviewers and readers to evaluate which analyses we planned and which we actually published.

## 3 Methods and analysis

### 3.1 Study design

The design is a triple blind randomised controlled trial, with two treatment conditions where therapists were randomised, 1:1 ratio, to either providing ICBT with a DST and a therapist manual (the DST condition) or providing ICBT as usual where only a standard manual is used (the TAU condition). Similarly, patients were randomised between the two conditions and then received 12 weeks of treatment in line with the procedures of that condition. Patients filled out online questionnaires at screening, pre-treatment, weekly during treatment, post-treatment, and at a 12-month follow-up assessment.

The trial has followed the guidelines of Good Clinical Practice (GCP) adapted for psychological treatments. This trial protocol was written in compliance with the guidelines for clinical trial protocols for interventions involving artificial intelligence, the Standard Protocol Items: Recommendations for Interventional Trials–Artificial Intelligence (SPIRIT-AI) extension (Cruz Rivera et al., 2020).

### 3.2 Randomisation

Randomisation of participant patients and therapists was done following two separate routines.

Patients were consecutively randomised according to a list with blocks of varying size, set up by an external partner, the Karolinska Trial Alliance (KTA), using the Alea Data Management randomisation system. The administrator managing the randomisation had no insight in the procedure or access to the list. The different sizes of the blocks were determined by KTA and unknown to the research team to prevent predictability.

Randomisation of therapists was done in cohorts with at least 2 therapists in each cohort. The trial coordinator first created subject IDs in the digital treatment platform P2 for each therapist and thus created a list of anonymous IDs that was ranked according to the therapists' treatment capacity (i.e. how many patients a therapist was expected to be able to treat). This was done to get as even a distribution as possible between intervention group and control group regarding treatment capacity. The anonymous ranked list of therapists for each cohort was then sent to KTA. After this randomisation was done by KTA for each cohort, using a web-based randomisation tool at www.randomization.com. KTA performed this randomisation of therapists in two separate ways depending on whether the list they received contained an even or uneven number of therapists:

1. If the number of therapists was even, the therapists were randomised in pairs, from the two with the most treatment capacity to the two therapists with the least capacity, so that one of each pair ended up in the intervention group and the other in the control group.
2. If the number of therapists was uneven, one of the therapists was first randomised to be randomised separately through a prepared block-randomised list with small blocks of varying sizes. The remaining therapists, which were an even number, were then randomised in the same way as described above.

To conclude, KTA made the required internal documentation and sent a pdf-document to the research team that described which group each therapist ended up in.

### 3.3 Blinding

Therapists, patients, and pre- and post-treatment assessors have been blind to what group therapists and patients were allocated to, consequently the study was triple blind. The informed consent explained for therapists and patients that they were going to be randomised and in very general terms informed them that the study was evaluating two slightly different models of how to structure the treatment process and supervise the therapists.

After randomisation patients were not given any further information about group allocation. Both groups of therapists were informed in a way that implied that they had been allocated to the experimental arm to further ensure their blindness and to avoid eventual biases connected to knowing being allocated to a certain arm.

For therapists, after randomisation those in the TAU condition were given a sham rationale for the study and were told that they would test new routines for how therapist and patient interact and that they as therapists would receive a more intense type of supervision than usual with more individual feedback on their messages. This was expressed explicitly when they started their first patient and were given feedback on the first messages. The purpose of this sham rationale, as stated earlier, was to give the impression that they were in the experimental group, and thus minimizing a risk of negative bias. However, the general treatment manual, clinical supervision and the clinical routines were the same for both treatment conditions and similar to routine care at the Internet Psychiatry Clinic (Titov et al., 2018). The therapists in the DST condition were informed that their group would test different routines for how therapist and patient interact, guided by a clinical DST that identifies patients at risk of treatment failure and a manual that instructed the therapists on how to act in relation to the specific patient. It is not possible to determine if the true study rational given to DST-therapists and the sham rational given to therapists in the control group affects expectations and engagement equally, but it at least ensures the control therapists are not discouraged or disappointed by being informed they are in a control group.

To enforce the blinding all therapists were required to sign a confidentiality agreement after randomisation, where they committed to not disclose information and details about the treatment condition in the study to outsiders, or other participants (therapists outside their supervision group as well as patients) in the study. The confidentiality agreement applies until the results of the study are published.

Taken together, the above precautions considerably reduce the risk of information leakage between groups and/or unintentional unblinding, but it still might occur in some cases. When detected, these will be reported.

### 3.4 Study setting, participants, and recruitment

The study was conducted at the Internet Psychiatry Clinic, an out-patient psychiatric clinic in Stockholm, Sweden, where patients since 2008 have been able to self-refer via the internet for an assessment for ICBT (Titov et al., 2018). The study has used ICBT-programs for depression, social anxiety, and panic disorder, which have been used and continuously evaluated since more than ten years at the Internet Psychiatry Clinic (Andersson et al., 2005; El Alaoui et al., 2015; Bergström et al., 2009).

The study evaluated both therapist behaviours and patient behaviours and outcomes, and hence they are both considered as participants, and were both give informed consent to participate in the trial.

### 3.5 Therapist recruitment

Therapists were recruited nationally in Sweden, being primarily students in the final year of the 5-year clinical psychologist program and intern psychologists (‘PTP-psykolog’), doing their first year of supervised practice before receiving their license, they all had basic education in CBT but little or no experience of ICBT. The choice of using novice ICBT therapists was motivated by two aspects; that this group were expected to be easier to recruit in large numbers and that it would reduce bias since they would have less preconceptions of how ICBT should be performed and for example might identify ICBT in the TAU condition as regular ICBT and might thus be less prone to believe in the sham rationale.

A majority of Swedish ICBT-studies have utilized therapists at similar levels and ICBT is a very structured clinical environment that offers good opportunities for supervision also of complicated clinical situations since the interaction with the patient is asynchronous. A study by Andersson et al. (2012) reports empirical evidence supporting that therapist's level of experience do not affect outcome in ICBT. In the current study the therapists received a high degree of clinical supervision, with both weekly supervision sessions as well as the possibility of on demand supervision. Adding to this the therapists were guided by detailed manuals and routines. Nonetheless, the lack of experience compared to for example therapists in regular care at the internet psychiatry unit could still be a factor that affects internal validity. Especially when adapting the treatment for at-risk patients since this more strongly relies on the overall clinical skills of the therapist than traditional ICBT-support does.

The students were recruited via messages sent to psychology student groups on social media platforms. Intern psychologists were recruited via emails to the national network for intern psychologist supervisors. Interested therapists were informed they would have the opportunity to participate in a free ICBT course and be supervised to learn and practice ICBT within the context of a clinical trial. After they had treated around five patients, they received a diploma. For therapists having treated the expected quota of five patients and who wished to continue as therapists in the study, they received a small monetary reimbursement for these additional patients.

All interested therapists were screened to ensure that they have the necessary level of CBT education by filling out an online questionnaire where they describe what clinical experience and CBT training, they had. They then went through general theoretical training for ICBT consisting of two 3-h online sessions. After this training a suitability test of each therapist was performed to ensure that they had reached an adequate level of understanding of ICBT. The test consisted of a written assignment with questions about the three ICBT programs and a demonstration of their clinical skills by writing replies to two messages from fictive ICBT patients. The study coordinator assessed all answers and decide if the quality was good enough for the therapist to treat patients in the trial. After training and testing therapists were randomised as described above.

### 3.6 Patient recruitment

The patients were recruited through the Internet Psychiatry Clinic's website and through advertising in social media. They were recruited nationally, and all patients were assessed via web-based screening questionnaires and those that fulfilled the inclusion criteria were also assessed in a structured 90-min diagnostic video interview, also informing about the treatment set-up, and assessing the patient's suitability and motivation for it. The assessment and the inclusion and exclusion criteria used mimicked the intake procedures at the Internet Psychiatry Clinic (Titov et al., 2018).

Inclusion criteria:

• 18 years or older.
• Social anxiety, panic disorder or depression diagnosis.
• Stable or no antidepressant medication for at least 2 months.
• No diseases, disorders, or substance abuse that required other, immediate attention (e.g., severe depression or suicidality).
• Available time for treatment and acceptance of its format.
• Proficient in Swedish.
• No ongoing CBT.

Exclusion criteria:

• Not available for assessment and diagnostic interview.
• Will not be in Sweden during the treatment period.
• Not able to receive text messages on a Swedish mobile phone.
• Not proficient in Swedish.
• No access to computer and internet.
• Not able to set aside about one hour a day to work on treatment.
• Bipolar disorder, if seeking depression treatment.

The psychiatric video assessments were conducted by licensed psychologist and intern psychologists, using the complete Mini-International Neuropsychiatric Interview, MINI (Sheehan et al., 1998) and the general inclusion criteria for assessing suitability for treatment. For an overview of the patient timeline schedule, see Fig. 3.

### 3.7 Interventions

All included patients were allocated to one of the three ICBT-programs for depression, social anxiety, or panic disorder (Andersson et al., 2005; El Alaoui et al., 2015; Bergström et al., 2009). Patients were allocated to a therapist belonging to the condition they had previously been randomised to. Therapists could be exchanged during treatment if they become ill, took time off or weren't able to fulfil the study because of individual reasons. The therapist that has had the most contact with a patient is considered the main therapist in the data analysis.

Patients logged in to the secure, online treatment platform to start the 12-week treatment program consisting of 10 modules. Each week patients read the text in the current module and worked independently with exercises as well as work sheets. When a module was finished patients filled out a homework report with structured questions about module content, exercises, treatment progress, and difficulties and how they had handled those. They then received an in-platform text message with feedback, encouragement, and help with problem-solving from their therapist who would then assign a new module. Besides the homework report they could also ask questions to the therapist via text messages in the platform and the therapists was obligated to answer within 48 h on weekdays. All patients also filled out the weekly symptom ratings for depression, including screening of suicidal ideation, and patients with social anxiety or panic disorder filled out their primary symptom questionnaire. The scores on these measures were shown in a graph in the treatment platform and was monitored by the therapist. The treatment platform activates a number of notifications, or ‘flags’ to alert the therapist to notable events. These flags are a basic function of the treatment platform and were shown to therapists in both the DST condition and the TAU condition. A flag indicating risk of suicidality was shown when a patient responded to item 9 on the Montgomery-Åsberg Depression Rating Scale-Self report version (MADRS-S; 41) with a score of 4 to 6. The platform also activated flags signalling that the patient had submitted a homework report or sent a message, when a patient had been inactive for >7 and/or 10 days, when there was seven days left of treatment, and when treatment ends.

All therapists had to have at least 1.5 years of basic CBT-training or corresponding level of knowledge. Therapists received two ICBT training sessions that were around three hours each. In the first training session they watched eight filmed presentations describing guided ICBT in general, how patients are assessed, its workflow from assessment to follow up, the treatment platform, how therapists should prepare and write messages to patients, how to handle patient inactivity, and how assessment of suicide risk is handled. They were also instructed read the ICBT programs for depression, panic disorder and social phobia. In the second training session the general therapist manual (supplement A) was presented, as well as a more detailed presentation of the treatment platform and all the routines described in the manual. After these two sessions all therapists had to do a suitability testing as described above.

Therapists were then randomised, and the two groups of therapists were separately introduced to the routines for respective treatment condition and then received their first ICBT patients. Therapists in the DST condition and the TAU condition wre supervised in separate groups. All therapists used a general therapist manual (supplement A), with detailed instructions on how to guide patients through the treatment program and how to manage different clinical scenarios, including recommendations on how much time to spend on each patient.

Clinical psychologist experienced in ICBT provided supervision via comments within the treatment platform and in weekly group supervision via secure video conferencing, including only therapists from the same treatment condition. Therapists could also consult the supervisor by phone if necessary. Therapists had to review a minimum of two messages with a supervisor before they were approved to continue more independently. Therapists were instructed to continuously follow the therapist manual that they have been trained in during the introductory course. In the therapist manual all clinical routines and scenarios were described. Supervisors monitored therapists' actions in the treatment platform to ensure that they adhered to clinical routines and guidelines for respective treatment condition. Supervisors were the same for both groups and were thus not blind but were instructed to always adhere to the separate manuals and guidelines for the two different groups and to always ensure that therapists were not mixed in the supervision groups.

### 3.8 DST condition

**Fig. 1:**
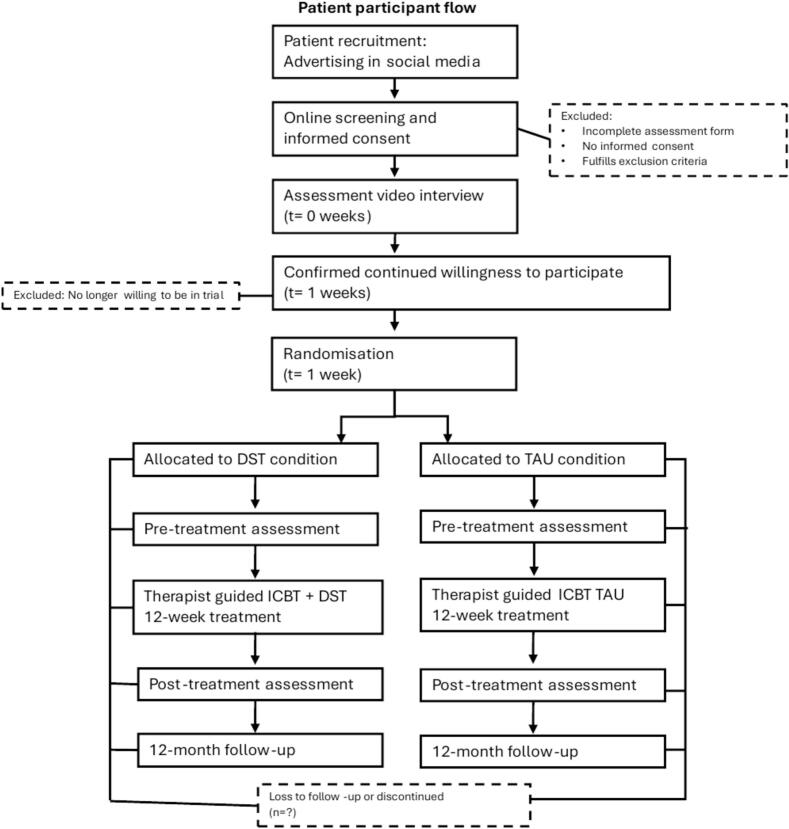
Patient participant flow chart.

Therapists randomised to the DST condition were guided by the general therapist manual, weekly supervision, and a specific therapist manual with instructions on when and how to act on the feedback from the DST. This DST manual also provided a general explanation of machine learning (ML) and how it had been applied to make the predictions in this DST. The primary indicator in the DST was a guiding recommendation where patients were classified into one of four colours that showed whether the patient's treatment was likely to be successful (green), likely to fail (light red), very likely to fail (dark red), or that the prediction was too uncertain to say anything about the outcome (yellow). Failed treatment was defined as being neither a responder (50 % symptom reduction from pre-treatment to post-treatment) nor a remitter (below remission cut-off score on diagnose-specific symptom scale at post-treatment). The DST also displayed graphs and information described more below. Fig. 1 shows the DST's graphical user interface.

#### 3.8.1 Actual symptom levels and predicted post-treatment level with prediction intervals

The first graph shows the actual weekly symptom scores that the patient has answered so far, as well as the decision support's latest, best prediction of the outcome at post-treatment. The prediction is displayed with two prediction intervals (90 % and 50 %) indicating how accurate the prediction is. If the patient has missed filling in a weekly estimate, a red cross is displayed. A dashed line marks the limit for “successful treatment” for the patient, i.e. the limit for the patient to become either a remitter or a responder, depending on what would first be reached.

#### 3.8.2 Activity index

The second graph shows with a grey horizontal bar how active the patient has been since the treatment started, compared to how active patients usually have been at that time in treatment. The grey bar thus represents at what percentile the patient currently is. The black dot, with safety margins of 50 % and 90 % shown with two shades of blue, is a prediction of the patient's total activity during the entire treatment at the end, also in relation to how active patients have been historically. The activity level is based on a combination of how actively the patient has worked with homework reports, sent free text messages to the therapist, and used worksheets, as well as amount plus frequency of log ins, in the digital treatment platform.

#### 3.8.3 Historical predictions of treatment outcome score by week

The last graph shows how the predicted treatment outcome has developed week by week, with safety margins (50 % and 90 % respectively).

The graphs are intended to provide background information to the therapist when assessing the patient's current condition and treatment progress. They are meant to be a complement to the main colour classification and do not provide specific guidance on how the therapist should act.

#### 3.8.4 Determining what colour is shown

To determine what colour a prediction of the post-treatment symptoms score should represent an empirical examination of the historical patients in the dataset the predictions was based on was conducted and the distribution of symptom outcomes for patients was examined. Those who were successful were classified as ‘green’. The remaining patients, who had failed, were split into a lower and upper half of the distribution based on symptom severity - where the more severe are classified as ‘dark red’ and the other as ‘light red’. Based on this empirical symptom distribution the symptom score equivalent of falling with the ‘green’ area, ‘light red’ and ‘dark red’ area was calculated. When a new prediction for a new patient is made, this continuous prediction score is checked against these cut-offs to determine the colour to be shown. The DST is calibrated on historical data to show a 50/50 ratio of green and red signals. This ratio closely corresponds to the overall rate of actually successful and failed treatments for these ICBT-program. This ratio has also been verified when testing the ML-model on historical patient data and is also in line with data from previous studies on these ICBT-programs (Andersson et al., 2005; El Alaoui et al., 2015; Bergström et al., 2009).

To determine when to show yellow for a patient, i.e. when the prediction is too uncertain, two different rules were used. During the first two weeks of treatment all patients are classified as yellow, to allow the ML-based prediction to become more stable and accurate, as shown in previous studies (Hentati Isacsson et al., 2024; Schibbye et al., 2014). Afterwards, to be classified as yellow the confidence interval of the prediction is checked, and if the interval crosses the decision boundaries for green or light red the patient is indicated as yellow. The confidence intervals of the prediction were chosen to diminish the number of yellow classifications over time. Going from an estimated 88 % yellow of historical patients at week three, to 0 % at week seven. Thus, the latest time in treatment yellow could be shown is at week six.

*The main guiding principles for the four different colour classifications are as follows:*

• **Green**: therapists should aim to limit the amount of time spent on these patients and to not do more than necessary by for example responding more concisely to messages from the patient, encourage the patient's independent work and to focus solely on the primary diagnosis for the treatment program. Therapists are instructed to spend, on average, 10 min per week on green patients.
• **Yellow**: therapists proceed doing treatment as usual and follow the guidelines in the general therapist manual. The guideline for yellow patients is to spend an average of 15 min per week on these patients.
• **Light red**: therapists must do a brief, delimited assessment of the patient's possible problems and difficulties with the treatment. This is done by sending a message about this to the patient and activating a digital questionnaire in the treatment platform, covering possible problem areas, for the patient to answer. The therapist then analyses the answers in this questionnaire and suggests an adapted treatment plan to the patient via messages in the treatment platform. If no obvious problems are identified, therapists are instructed to wait with adaptations, given that the supervisor agrees. However, if the patient is still light red after another 1–2 weeks the therapist must do at least one adaption. Light red adaptions should be limited concerning how time consuming they are for the therapist.
• **Dark red**: therapists must contact the patient as quickly as possible to do a structured telephone assessment of the patient's possible problems and difficulties with the treatment. The therapist analyses the answers from the assessment and suggests an adapted treatment plan for the patient. The therapist also checks this plan with the clinical supervisor. If it is difficult to implement the telephone assessment therapists are instructed to get supervision to find alternatives, which for example could be to activate the digital questionnaire in the treatment platform. Dark red classification implies that the therapist, regardless of his or her own clinical assessment of the patient's situation, must make an assessment and some form of adaptation. Dark red adaptations are allowed and expected to be more time consuming than light red adaptations.

For light and dark red patients, both the digital questionnaire and the telephone assessment were presented as a routine check-up that was done during treatment with the purpose to see how things are going and to identify possible problems and obstacles. For the telephone assessment the therapists were instructed to follow a semi-structured interview guide which focused on possible problem areas like technical/practical difficulties, time management, organising and working with homework, reading module texts and messages, understanding the treatment rationale, contact with the therapist, circumstances, or events outside of treatment, the patients' motivation, and other possible obstacles to working successfully with the treatment. The interview guide does not mention the DST, patient symptom levels, specific symptom questionnaire items, or the colour signals, and these aspects are not referred to as the reasons the patient is contacted. For a more detailed description of the telephone assessment, see supplementary materials: C. *Interview Guide - Dark Red*.

The therapist's clinical assessment of whether the patient needed adaptions was allowed to override the colour recommendations from the DST and the manual. For example, a dark red patient could be assessed to be very active and motivated in treatment, doing all exercises correctly and not showing any need for extra support, then the therapist (after consulting the supervisor) let the patient continue treatment according to plan, but documented this decision and monitored the patient's development.

**Fig. 2:**
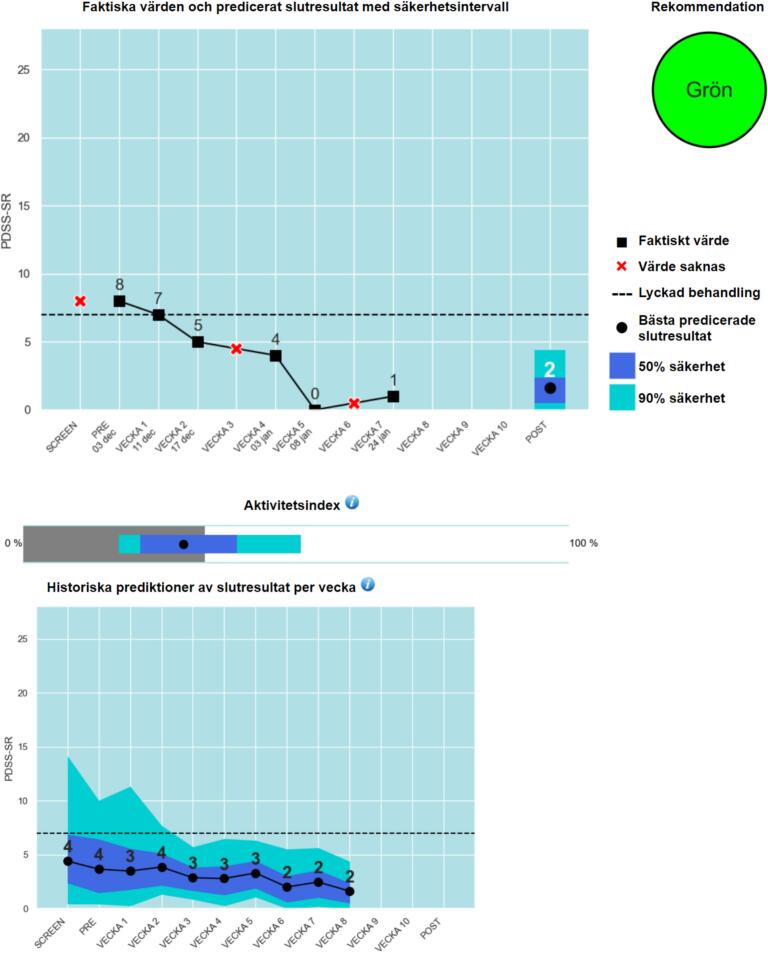
DST Graphical user interface.

**Fig. 3:**
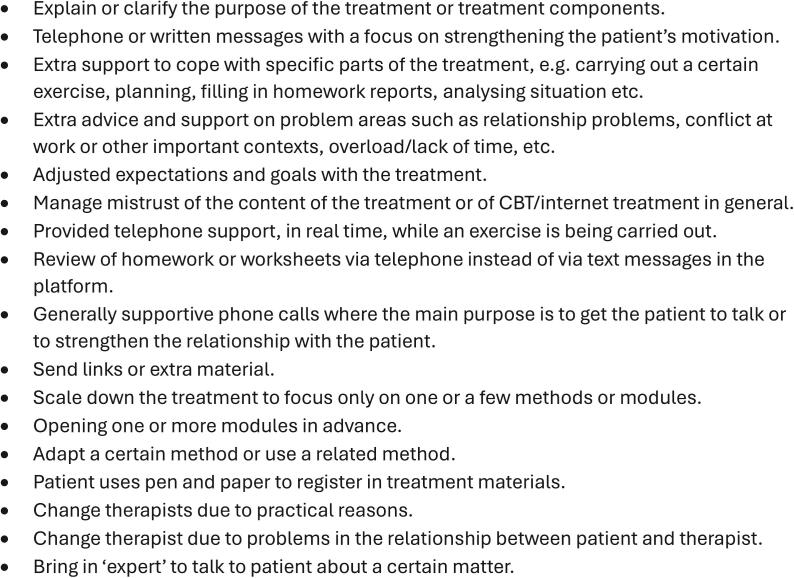
Examples of actions when adapting treatment for light and dark red patients.

To help therapists decide on suitable adaptions, the DST manual provided a number of suggestions, explanations, and examples. For green, i.e. not-at-risk patients, this included for example tips on how to give relevant and empathic, but briefer, answers to their homework reports. Fig. 2. presents a list of suggested adaptations for at-risk patients, and the complete description can be seen in the supplementary material: C. *Interview Guide - Dark Red*. To avoid affecting how patients answer the self-rated primary outcome, the therapists do not mention or discuss the score or answers on individual items on these when they assess the patient or suggests adaptions.

#### 3.8.5 Training of the machine learning model

The machine learning model used for the DST has been trained on >6000 historical patients from the same clinical and technical setting, with the goal to at any time during treatment predict if the treatment for a certain patient would fail (being neither a responder or remitter, defined in the same way as the corresponding outcome measure in the current trial, described below) or succeed. The model is installed and run on the same server responsible for hosting the trial treatment and applied each night on all active patients active in treatment. The result of the predictions for each patient is provided to the DST's graphical interface coded and set up in a web server gateway interface for python using uWSGI. The therapist get access to the DST through the same login session as the treatment platform P2.

The version of the machine learning model used was a second iteration of the online DST, and the first trained with the current parameters. It's trained was complete at 2021-05-21, before the trial started. The model was based on work in a previous study (Hentati Isacsson et al., 2024) where the authors examined how choices in relation to three aspects: (1) variable selection, (2) missing data management, and (3) algorithm selection impacted model performance. In this study a total of 1680 predictive models were built, analysed, and compared. The model used for the DST in the current study was selected from this group of models by primarily looking at consistency of the models' performances across six-time points of prediction and the predictive balanced accuracy.

Based on the initial model building study (Hentati Isacsson et al., 2024), input data used as predictors in the DST model came from two categories of variables: self-rated assessments from patients and different types of indicators of patient activity during treatment, for example meta-data from log ins, messages, and homework. Variables used in the deployed model were manually selected, since the previous study found this to generally be superior compared to PCA-type variable selection, with a focus on prediction in relation to outcome and aimed to limit the number of features to avoid overfitting. The selection was done by including a) variables that indicate activity in the treatment (e.g. what day of the week patient was active, number of messages, duration to fill in questionnaires), b) variables important to symptom outcome (e.g. the sum of the primary symptom questionnaires), and c) demographic variables.

The model used the designated predictors as previously mentioned and imputed missing values using Missforest in Python. It was trained using a random forest algorithm based on the hyperparameter tuning done during the initial study (Hentati Isacsson et al., 2024). Random forest was chosen for its general accuracies as well as generating adequate prediction intervals in the initial study, where the balanced accuracy was around 74 % and was evaluated using both 10-fold cross validation and a 10 % holdout test set. With the symptom variables having the largest influence on predictions. For a more detailed description of the background to this model development and feature selection see Hentati Isacsson and colleagues (Hentati Isacsson et al., 2024).

List of included features in the DST ML-model and when they are measured:

• Primary symptom measure (screening, pre-treatment, and weekly)
• Secondary symptom measure (weekly) (weekly MADRS-S for patients with panic disorder and social phobia, and PHQ-9 at screening and pre-treatment for patients with depression)
• MADRS-S items 3 (sleep) and 9 (suicidality) (weekly)
• Which treatment the patient is in
• Time in milliseconds to fill out symptom scale (weekly) - both primary and if applicable secondary symptom measure
• Day of week filling in weekly measures
• Time of day filling in weekly measures
• Patient reporting having completed a homework activity (one or more activities depending on module, weekly)
• Number of submitted homework reports (weekly)
• Number of written characters in homework report (weekly)
• Number of messages sent (weekly)
• Length of messages sent (weekly)
• Number of therapist messages (weekly)
• Length of therapist messages (weekly)
• Number of characters in worksheets (weekly)
• Number of words in worksheets (weekly)
• Number of logins (weekly)
• Login weekday (weekly)
• Login time of day (weekly)
• Number of days with at least one login (weekly)
• Number of days without login so far
• Longest period of days without login so far
• Duration spent logged in on platform, time logged in (weekly)
• Which year the treatment started
• Which week number of the year the treatment started
• Age
• Sex
• LSAS-SR item 17 a (taking a test - anxiety) and 17 b (taking a test - avoidance) (pre-treatment)
• Treatment credibility scale (week 2)

### 3.9 TAU condition

Therapists randomised to the TAU condition were only guided by the general therapist manual, weekly group supervision and individual on-demand supervision. Therapists would, through the manual and in supervision, be encouraged to assess how the patients were doing and to monitor their progress. They were recommended to spend an average of 15 min per patient and week. The general obligatory routine for the therapists was to log in to the platform a minimum of three times per week and to act on the different general flags displayed for their respective patients, as described above. Therapists were also instructed to regularly monitor patient progress in the treatment platform, in a graph that showed the patients weekly symptom ratings. For a more detailed description see the translated version of the general therapist manual in the supplementary material (supplement A).

As noted above therapists in the TAU condition were given a sham study rationale to give the impression that they were in the active experimental group and that the trial was evaluating the effect of their specific therapist manual and a more intensive type of supervision. This procedure was expected to lower the risk of negative bias (similar to nocebo effects for patients) due to therapists suspecting they were in the control group.

### 3.10 Monitoring and addressing potential adverse events

During the trial, there were routines to identify and act on deteriorations in psychological well-being, as well as suicidality risk. These routines were the same as the routines used in regular care in the Internet psychiatry unit. Depending on how acute the problems were, these events were handled by the trial coordinator or therapists within the project or referred to regular psychiatric care or emergency care at the patient's place of residence according to predetermined routines. The therapists also had the support of an experienced clinical supervisor to make decisions in such situations. A specially designated medical doctor in charge was also available for consultation.

These routines are described in the general therapist manual. The trial coordinator monitored risk of suicidality during weekdays in the treatment platform where warning flags were activated for patients both in screening and during treatment if they responded to item 9 on the MADRS-S scale with a score of 4–6, indicating elevated risk. The coordinator then contacted the patient by telephone for a semi-structured suicide risk assessment interview. This was complemented by therapists reporting when they read messages from patients with content that indicates risk of suicidality. Rapid deterioration was reported and handled at the weekly supervision sessions. If therapists assess that deterioration was acute, they were instructed to contact the trial coordinator or their trial supervisor for further assessment and action.

### 3.11 Outcomes and measurements

Patients background data, primary and secondary outcomes, and process and therapist related measures were assessed in the online treatment platform.

Patients logged in to the treatment platform when they first registered for the study and filled out the screening questionnaires. After screening patients were contacted for a diagnostic video assessment interview. Included patients answered the pre-treatment questionnaires when starting treatment, before getting access to the treatment modules. Some questionnaires were specific for the three different treatment programs (depression, panic disorder or social anxiety) and some were administered to all patient participants. During the 12 treatment weeks, patients filled out the weekly questionnaires in the online treatment platform and some questionnaires were administered at specific weeks during treatment (specified below). The post-treatment questionnaires were activated in the online treatment platform 7 days before the treatment ends, to decrease the risk of data attrition. Long-term follow-up self-assessment is performed 12 months after the pre-treatment measure.

All patients were contacted for a telephone assessment after the treatment was finished. This was done by blind assessors. The main purpose was to assess clinical aspects; how the treatment had gone, assess use of other treatments during the trial period, and need of further treatment. The assessors also collected the post-treatment ratings via the telephone if the patient had not done this online in order to lower attrition of the post-treatment measure and according to a previously tested routine (Hedman et al., 2013). Patients in need of further treatment will be guided to where they can seek the proper type of care.

### 3.12 Primary outcome measures

Primary outcome is symptom change during the 12-week treatment, represented by the one of the three below listed patient-rated, diagnose-specific symptom scales being specific for the ICBT-treatment a patient received. They are all measured at screening, pre-treatment, each week in treatment, post-treatment and at 12-month follow-up.

• Montgomery-Åsberg Depression Rating Scale-Self report version (MADRS-S) (Bondolfi et al., 2010) is the primary symptom measure for the depression treatment. It is specially developed to be sensitive to change, and higher scores (0–54) mean more symptoms of depression.
• Panic Disorder Severity Scale - Self Rated (PDSS-SR) (Furukawa et al., 2009; Svensson et al., 2019) is a self-report scale for panic disorder that has been shown to be sensitive to change with treatment. Is in this study the primary symptom measure for panic disorder, where higher scores mean more panic disorder symptoms. Min - Max score = 0–28.
• Liebowitz Social Anxiety Scale, self-report (LSAS-SR) (Fresco et al., 2001; von Glischinski et al., 2018) is a self-rated scale for assessment of social anxiety disorder, and it has two subscales for fear and avoidance. Is in this study the primary symptom measure for social anxiety, where higher scores mean more symptoms of social anxiety. Min - Max score = 0–144.

### 3.13 Secondary outcome measures

• Failed/Successful Treatment. Failure is defined as being neither a responder (50 % symptom reduction from pre-treatment) nor a remitter (<11 on MADRS-S (Bondolfi et al., 2010); <8 on PDSS-SR (Svensson et al., 2019); or < 36 on LSAS-SR (Fresco et al., 2001)). This is measured from pre-treatment to post-treatment. Also, the separate dichotomous outcomes of responder and remitter will be measured and reported.
• Euroqol (EQ-5D) is a short questionnaire for measuring health related quality of life (Burström et al., 2014).
• WHO Disability Assessment Schedule (WHODAS) is a self-rated measure of daily functioning and an assessment instrument for health and disability available in 36- and 12-item versions. We will use the 12-item version (WHODAS-12) (Axelsson et al., 2017).
• Number of treatment modules each patient has completed. Coded from the treatment platform throughout treatment from pre-treatment to post.
• Four questions measuring treatment adherence, belief in treatment and knowledge, are measured four times during treatment (week 3, 6, 9, and post-treatment).
  The first question asks how much the patient has worked with their homework and if they have done it in line with the given instructions, during the last two weeks, with the scores 0 (none or very little), 1 (tried but unsure how to do it), 2 (partly in line with instructions or used other methods than those in treatment), 3 (worked with homework from most recent module only or from previous modules only), and 4 (worked with homework both from recent and previous modules).
  The second question asks if they have been able to work with the treatment as much as they wanted or planned from 0 (No, did not want to) to 4 (Yes, with a good margin). A combined index for adherence is created by adding these two scores from the first two items from all four measurement points. Missing data will be defined as 0 activity/adherence.
  The third question asks how much the patient believes that the treatment suits them and how much their situation has changed during the recent week, from −2 (believes less) to 0 (unchanged) and gradually up to 3 (believe much more). The score for belief in treatment is the sum of all measurement points, where missing data count as 0.
  The fourth question measures how much the recent week in treatment has changed the patients' knowledge about their problem/diagnosis, how they perceive their problems, and how the problems can be managed, rated from 0 (unchanged) to 4 (changed very much).
  For details see supplement D.
  For details see supplement D.
• The Client Satisfaction Questionnaire 8 items version (CSQ-8) (Attkisson and Larsen, 2012) will be used to measure patients' satisfaction with the care received. Each item is rated on a 4-point Likert scale with scores ranging from 1 (None of my needs have been met) to 4 (Almost all of my needs have been met). The total score range is 8 to 32 where a higher score indicates a higher degree of satisfaction.
• The Internet Psychiatry Clinic's standard patient evaluation questionnaire (version 3), 17 items covering patients experience of accessibility of the treatment, how they in general have been received, how actively they have worked with the treatment, if they have encountered problems with the treatment, if they have received any other treatments during the study period, if the treatment program was lacking something, and what they experienced as most helpful in the treatment program. Measured at post-treatment.
  **To view questionnaire, see**supplement D.
  **To view questionnaire, seesupplement D**.
• Trial specific extra patient evaluation questionnaire with questions covering the patients experience of the treatment in general, the working alliance with the therapist, and the treatment adaptations. Measured at post-treatment.
  **To view questionnaire, see**supplement D.
  **To view questionnaire, seesupplement D**.
• Patient-rated Treatment Credibility Scale (min - max = 0–50, higher scores indicate higher perceived treatment credibility) (El Alaoui et al., 2016), consisting of five items covering how logical the patient perceives the treatment, how successful they think it will be, if they would recommend it, and how improved they expect to become from the treatment. Measured at week 3 in treatment.
• Number of Adver Events and Serious Adverse Events reported by patient. The patients are asked if they have experienced any adverse events from the treatment and to describe these. Number and degree of Adverse Events are also assessed by blind assessors in the telephone assessment after the treatment is finished. Measured at post-treatment.
**For details see** supplement D **.**
**For details see supplement D.**
• Patient reported need for further treatment. The patients are asked if the current treatment has been sufficient, if they still need treatment for the problem, they sought help for, if they plan to shortly seek treatment for this problem, and if they plan to shortly seek treatment for any other problem. Comparable questions are asked by blind assessors in the telephone assessment after the treatment is finished. Measured at post-treatment.
**For details see** supplement D **.**
**For details see supplement D.**

### 3.14 Treatment process and therapist perspective measures

• Numbers and length of messages sent to and from the therapist. Logged digitally in the treatment platform. Length of messages is measured by number of characters.
• Number of mobile text messages sent from the treatment platform or from external mobile device. Logged digitally in the treatment platform.
• Time spent by therapist on each patient. Logged digitally in the treatment platform at each interaction with patient and summed at post-treatment. The time logs will be checked for possible outliers that have been incorrectly logged. The corrected time will be based on a calculation of time from logged activities and messages.
• Number, length and type of telephone calls from therapist to patient. Logged digitally in the treatment platform at each interaction with patient and summed at post-treatment.
• Number of letters sent to the patient. Logged in the treatment platform.
• Time efficiency: Degree of change on the primary outcome divided by therapist time spent on the patient. Measured throughout treatment from pre-treatment to post.
• Therapist CRF notes on what treatment adaptions they have made and if they have used supervision for this. This is noted generally for all patients by therapist in the TAU condition. Therapists in the DST condition make more specific notes of type of adaptations and if they have used supervision, connected to the different colour recommendations they have received for the patient.
• Therapist questionnaire, DST condition and TAU condition versions. Both versions have 17 items. Item 1–7 measures therapists general experience of using the DST or the general manual only (i.e. not related to a specific patient), and how they experience trust, usability, understandability, and guidance concerning the DST or the general manual only. The questionnaires also measure therapists experience concerning perceived helpfulness, to what extent they feel that they have learnt anything new, and their overall experience of supervision, clinical routines, and guidance of their clinical decisions. This is measured either when the therapist has fully treated 5 patients or when the therapist leaves the study (if that happens before treating 5 patients and has treated at least 1 patient). **For details see** supplement D**.**
  Therapist questionnaire, DST condition and TAU condition versions. Both versions have 17 items. Item 1–7 measures therapists general experience of using the DST or the general manual only (i.e. not related to a specific patient), and how they experience trust, usability, understandability, and guidance concerning the DST or the general manual only. The questionnaires also measure therapists experience concerning perceived helpfulness, to what extent they feel that they have learnt anything new, and their overall experience of supervision, clinical routines, and guidance of their clinical decisions. This is measured either when the therapist has fully treated 5 patients or when the therapist leaves the study (if that happens before treating 5 patients and has treated at least 1 patient). **For details see** **supplement D.**
• System Usability Scale (SUS). Measuring usability of the DST from the therapist perspective (not related to a specific patient). Min - Max score = 10–50. Measured either when the therapist has fully treated 5 patients or when the therapist leaves the study (if that happens before treating 5 patients and has treated at least 1 patient).

### 3.15 Data management and monitoring

Data is processed for research purposes according to the EU Data Protection Regulation (GDPR) based on the legal basis of performing a task of public interest. All data are stored in the secure P2 system, i.e. the web-based platform providing the treatments and the questionnaires to patients. Patients' self-reports are included in the system and directly stored. Data from clinician's assessments, interviews, and logs of treatment processes (for example number of phone calls made) are either entered directly in P2 or first on paper and then entered in P2. Therapists' questionnaires are also administered and stored in P2. P2 questionnaires have built in data validation ensuring a complete data entry for each instrument used. Free text data are analysed qualitatively and categorized in themes by the research group and/or assistants when relevant.

Login to P2 is done via a two-step authentication for patients, therapists and researchers. Participants cannot access answers to their own or other participants ratings through the system. All communication between participants and therapists takes place within the secure platform. All information in P2 is stored on a server that is in the same premises as the regular clinic servers with electronic medical records and uses the same protection against data breaches as these. In P2, each participant is automatically assigned an ID number and when the data is retrieved for statistical analysis, only this ID number is extracted, to make the data pseudonymized (still to be considered as personal data). The data in P2 will thus act as a code key and it will not be destroyed. Exported data is stored on the same secure server or, after a personal data processor agreement has been concluded, on a server with equivalent security at the personal data processor.

Only therapists, researchers and research assistants involved in the project have access to personal data, regardless of whether it is in P2 or has been exported from P2. In some cases, personal data is also managed by research collaboration partners after the cooperating organization has entered into a personal data processing agreement with KI where handling of data is regulated. In some cases, in addition to pseudonymisation, the data will also be modified so that it cannot be traced back to an individual at all, partly by removing ID codes but also by removing other data that can indirectly identify a person, e.g. visit dates. This type of data is no longer personal data and can be used in accordance with the principles of so-called Open Data, and for example be published so that other researchers can access it.

No Data Monitoring Committee was used since this is not required when the intervention is not testing a pharmacological drug or a medical device, according to Swedish law. Due to the expected low level of risk and severe Adverse Events, no interim analysis or pre-defined stopping rules are used.

The machine learning model used in the DST underwent testing and performance analytics before trial start. This was done within the testing and performance analytics concerning a range of different models applied on the historical patient data set used for training, which were used to investigate technical errors (Hentati Isacsson et al., 2024). Furthermore, since the model runs daily, trial therapist as well as clinical supervisor of the trial have a possibility to daily investigate the performance of the model.

### 3.16 Power and sample size calculation

Before the start of this study, while conducting the power analysis, our basis for estimating the expected effect size was a previous ICBT-based proof-of-concept trial where the primary analysis corresponded to ours, i.e. where differences in pre-post changes in symptoms for patients at-risk was compared, an effect of Cohen's d = 0.59 was found (Forsell et al., 2019). Allthough similar in many aspects, that trial evaluated ICBT for patients with insomnia and used another type of outcome prediction, and might not be fully generalizable to depression or anxiety patients in the current study. We found no other similar ICBT-studies, and thus looked beyond the ICBT context and alsobased our estimation on a meta-analysis by Lambert et al. (2018) examining the impact of measuring, monitoring, and feeding back information on client progress to clinicians while they deliver traditional psychotherapy. This meta-analysis reported a small effect size of 0.14 for the whole sample of patients, a larger effect (0.33) for feedback on at-risk patients, and a still larger effect (0.49) when clinical support tools, directing clinician problem solving with at-risk patients, was provided to therapists. The latter best mirrored our primary analysis with a focus on at-risk patients and using a clinical DST guiding therapist problem solving and treatment adaptations. Taken together, we estimated the effect for our primary analysis in the current trial to be 0.50. A power of 80 % and an attrition of 20 % at post-treatment would require 158 at-risk patients, and thus 316 in total given that 50 % of patients would be identified as at-risk, as is expected due to previous distributions of final outcomes and how the DST is calibrated. To increase the power also for secondary measures, the aim was to include 350 patients. If this number was reached when the patient quota for all included therapists was not full or while an ongoing recruitment period (spring or autumn) had not reached the end, more patients would be included to fill each therapist quota or until end of the recruitment period, if project resources allowed it. This was motivated by the uncertainties behind the power analysis and means that the final number of included patients could exceed 350 to further increase statistical power. After the estimation of the expected effect size in our power analysis was set, a later multilevel meta-analysis by de Jong et al. (2021) came out. This meta-analysis showed a similar pattern as the meta-analysis by Lambert et al. (2018), with a smaller effect size for the at-risk subsample (0.17) and a larger effect (0.36) for studies using clinical support tools. The latter effect size indicates that our expected effect size could be overestimated. However, with the above aim to strengthen statistical power for our secondary analyses, we had the opportunity to include in total 401 patients.

### 3.17 Data analysis plan

The choice significance level will be 0.05 in all analyses. Assumptions of models will be checked, and possible violations will be reported and amended.

### 3.18 Definition of at-risk patients

Patients in both conditions will be classified as being at risk if they at any time during treatment have been, or for the TAU condition ‘would have been’, classified as Red (light or dark) by the DST. However, since the prediction of treatment outcome and the colour classification is updated every night, it is theoretically possible that a patient are Red for just one, or a few days, and that the therapist are not logged in during these days. The DST logs when a therapist views the prediction and classification of a certain patient. Thus, to be classified as an at-risk patient in the DST condition, the system log must confirm that the therapist actually saw the red colour, otherwise the patient is classified as not-at-risk. If the number of unseen red classifications exceeds three then the pattern for when the unseen Reds appear will be examined. If then for example weekends or red periods of just one day clearly define unseen Reds, that will be used a rule to also define the corresponding Reds in the TAU as not-at-risk patients. If a systematic pattern cannot be found, or if the number of unseen Reds in DST are three or less, then all patients in TAU having been Red at least one day will be classified as at-risk.

To enable a sensitivity analysis, an alternative classification of ‘at-high-risk patients’ will be made using the same principles as above, but only including patients at any time being classified as Dark Red.

### 3.19 Primary analysis of patient outcome

The primary outcome measure is change in respective diagnose-specific measures in each ICBT-program from the pre- to post-treatment measure, including all weekly measures. To allow outcome data from all three measures to be included in the same analysis the raw scores are converted to the percent of each scales maximum score. Primary analysis will be made on an intent-to-treat basis (or rather an ‘intent-to-use-the-DST basis’) where all patients that are randomised to the DST or TAU condition and are classified as at-risk patients (see above) are included. Missing data will be managed by multiple imputation using as many variables as possible after correcting for multicollinearity, and in line with existing recommendations (Van Buuren, 2018) an HLM-model will then be fitted. This model will be fitted with all measurements of symptom data from pre to post and including the grouping variable for treatment condition (DST or TAU condition). Since patients are grouped under therapists, even though they are independently randomised, the model will be nested in three levels: time, patient, and therapist. The model will be fitted with a random intercept and slope for time, and we will also examine if adding a quadratic effect of time (time X time) would increase fit and then use that model. The primary analysis and significance test evaluating the effect in the DST condition will be the interaction of Time X condition. However, if it is found that the best fit includes an interaction where the quadratic effect of time differs between the conditions (condition x time x time), i.e. if the curvature of the trajectory of the DST condition and the TAU condition are significantly different, then the basic interactions effect (time x condition) will be very difficult to interpret and instead the estimated difference between conditions at the post-measurement will be used as primary analysis.

#### 3.19.1 Sub-group and sensitivity analyses related to the primary analysis

Towards the end of treatment, the therapist will have little time to do an extra assessment and implement treatment adaptions for patients identified as at-risk, due to the strict time-limit of ICBT. This will most likely affect the possibility for the DST condition to show superiority over the TAU condition for at-risk patients identified late in treatment. This is seen as an innate limitation in the current procedure for ICBT and thus all at-risk patients are included in the main analysis (above), but to examine this effect a sub-group analysis excluding all patients identified as at-risk later than 2 weeks from the end of treatment are also performed. Another sub-group analysis will define at-risk patients more conservatively by only including those becoming Dark Red (i.e. higher risk of treatment failure then primary analysis where also Light Red are included). Also, sensitivity analyses are made by recalculating the primary analysis without the therapist as a nested level, and by excluding all unseen Red patients in the DST condition instead of classifying them as not-at-risk.

### 3.20 Secondary analyses of patient outcomes

Below is a description of how each secondary hypotheses or research question related to patient outcomes will be analysed.

• The DST condition will decrease the number of failed treatments among patients identified to be at risk.
  The imputed dataset pre- and post-measures will be used to define each patient as failed or successful, and a chi-2 test will then be used to test differences between the DST condition and the TAU condition.
  Two sensitivity tests are performed on observed data, with and without replacing missing post-measures with the last know symptom levels.
  The same analysis is performed for the dichotomous outcomes responder and remitter respectively.
  The DST condition improves everyday functioning, health related quality of life, patient satisfaction, number of Adverse Events (AE) experienced by the patient, and need for further treatment.
  HLM models corresponding to the one used for the primary outcome will be used on continuous measures collected at pre and post (WHODAS, EQ-5D) and between groups *t*-tests or corresponding non-parametric tests for CSQ, number of AE and need for further treatment.
  The description of Adverse Events will also be analysed qualitatively into different themes and categories and descriptive statistics for those are presented.
  The DST condition increases the adherence to treatment among patients at risk.
  t-tests or corresponding will be used to compare the number of finished modules, the index created from the two patient-rated questions about adherence measured during treatment, and the post-measures of how much of the treatment texts they have read and how much of all homework they worked with.
• The DST condition improves levels of symptoms, functioning, patient satisfaction, Adverse Events, interaction, and adherence when all patients (also those not-at-risk) are included.
  The same types of analyses that is described for at-risk patients will be used, but in this case all patients, also those not-at-risk, will be included in analyses.
  Differences in all outcome variables will be explored for patients not-at-risk, to examine indications of negative effects of being in the DST condition for this group.
  Same analyses as above, but for not-at-risk patients only.
  Evaluation if the DST condition effect differs between ICBT-programs (i.e. for depression, social anxiety disorder, or panic disorder), and between patients of different levels of problem severity at intake.
  To evaluate this, the main analysis is complemented with two dummy variables coding for the three different ICBT-programs. Also, six density curves (2 study conditions x 3 ICBT-programs) are made for visual inspection with CI-95 %, and corresponding för severity levels.
• For patients in the DST condition, the decrease of symptoms will be faster during the period from the time-point they are identified as at risk and to the post-treatment measure, compared to their period from pre-treatment to the identification point and compared to the rate of symptom decrease from pre to post for all identified at-risk patients in the TAU condition.

HLM model with two timepieces and interaction with treatment condition will be used for the at-risk patients. A coding scheme with separate slopes for the first respective the second time period will be used. The first timepiece will represent time before being judged as at risk, and the second timepiece will represent time from being judged as at risk to post-treatment. Both timepieces will be tested for interaction with condition, to investigate if change differs in any of the time periods depending on treatment condition. Additionally, the coefficients will be compared using a z-test to see if improvement in symptoms differ between the two time periods.

• There will be a trend showing that the effect (on symptoms) in the DST condition (compared to the TAU condition) will be largest for at-risk patients detected earlier in treatment and then decrease the later in treatment the detection occurs, since there is less time for treatment adaptions to have effect due to the ICBT-programs being restricted to 12 weeks.
  To examine the effect of the DST condition in relation to when a patient is identified as at-risk, the between-group effect size (Cohen's d) from the last measure before the point of identification to the post-measure for all patients identified from week three (since week 1–2 always shows yellow) and each week forward will be calculated with CI-95 % and presented in a table and/or graph for mainly visual inspection.
• In the DST condition, at-risk patients will decrease their symptoms as much as patients not-at-risk but to a lesser extent be defined as remitters after treatment, while in the TAU condition the at-risk patients will improve less on both these measures than patients not-at-risk.
  Two HLM-models, one for the DST condition and one for the TAU condition, similar to the one used for the primary analysis will be used with a grouping variable for if patients are at-risk vs not-at-risk to explore if the interaction ‘risk-definition’ x time differs. Also, chi-square tests to explore if number of remitters differ between at-risk and not-at-risk patients will be made separately for the DST condition and the TAU condition.

### 3.21 Process and therapist related analyses

• The DST condition increases the therapists' amount of interaction with patients at-risk and time spent on patients at risk and decrease it for not-at-risk patients.
  Independent *t*-test will be used to compare the DST condition to the TAU condition regarding number and length of messages to and from the therapist, mobile text messages, and number and length of video/phone calls for at-risk patients and not-at-risk patients respectively.
• Therapists in the DST condition will overall be more time efficient, defined as the ratio of ‘decrease in symptoms / therapist time spent per patient’.
  The analysis will be made on all patients and the ratio will be calculated for each patient by using the pre-post difference in primary symptom (where missing post-measures are imputed) and the total time the patient's main therapist and other therapists have spent on the patient. An independent *t*-test will then be used to compare the ratios between the DST condition and the TAU condition.
• Therapists in the DST condition will perceive the DST in combination with the therapist manual as more trustworthy, understandable, useful, and helpful than therapists in the TAU condition will perceive the therapist manual only. This is measured in the Therapist questionnaire where items covering these areas, Likert scores are summed.
  Description of therapists' overall experience of supervision, clinical routines, and guidance of their clinical decisions and if this differs between the two conditions.
  This is measured in the Therapist questionnaire where items covering these areas, Likert scores are summed. *t*-test or corresponding are used to test differences.
• Description of adaptations therapists have used for at-risk patients in the DST condition and if they differ from at-risk patients in the TAU condition and from not-at-risk patients. This is taken from the therapists CRF notes made at the end of treatment.
  Evaluate if there for all patients is a therapist effect on the primary outcome.
  How much of the explained variance that is due to therapists in the primary analysis model is used to evaluate therapist effect. This will also be examined for the DST and TAU conditions separately.
  Is there a training and experience effect where therapists' early patients benefit less than later patients.
  The effect for each patient is defined as the pre-post difference in primary symptom (where missing post-measures are imputed). Each patient also receives an order number depending on if he or she was the first, second etc. patient for their main therapist. The correlation between these variables will then be calculated. However, some therapists could have markedly more patients than others and would then have a very strong influence on the correlation. Thus, outliers in the model will be examined if some therapists have a markedly higher number of patients than the mean and sensitivity analyses excluding or correcting for those will be made.
• Performance of the predictive model the DST is based on and exploration of new models.
  Data from the TAU condition will be used to calculate the balanced accuracy with CI-95 % of the predictive model that the DST is based on, to compare its performance on this new sample of patients to the historical groups of patients it was trained on to predict post-treatment symptom scores. Also, specifically patient data in the TAU condition will be used to explore new predictive models and evaluate single variables as predictors.

### 3.22 Patient and public involvement

During the spring of 2020, the authors developed a first version of a DST giving ICBT therapist at the Internet Psychiatry Clinic feedback on their patients' predicted end state. We involved the users (therapists) in the development process and in a pilot trial with actual patients and evaluated its acceptability and perceived usefulness before finalizing its design (manuscript in preparation).

Patients were not involved in the design, the recruitment, or the conduct of this study since they were not the primary users of the evaluated DST. However, in future trials also the patients' general perspectives and attitudes in relation to using AI/Machine Learning to guide psychological treatment should be included and considered, as well as their more specific input on how to design DST:s and the health care routines where these new digital applications are used.

## Trial registration details, ethics, and dissemination

The ethics application process in Sweden is distinctive, as it is managed by the governmental body SERA rather than local or regional committees at universities. The application is archived in the state archive and can be sent upon request, thereby serving as a form of preregistration and thus listed in the abstract as a preregistration. The version of the study protocol registered with SERA (approved 25/11/2020, #2020–05772) is identical to the Clinical Trials registration (approved 04/04/2022, #NCT05321628) in terms of hypotheses, with only minor differences in wording and structure. The power analysis in the SERA was subsequently updated in Clinical Trails due to the discovery of an error in the SERA protocol, where it had been based on an incorrect historical effect size and on all patients in each arm instead of only the at-risk patients as specified by the primary hypothesis. Importantly, no data from the 13 patients enrolled between the study's commencement on December 3, 2021, and the approval of the Clinical Trials registration on April 4, 2022, was utilized in the power analysis or for any other purpose. Hence, even though the Clinical Trial registration could technically be considered a retrospective registration, the issues related to that is handled by the SERA-preregistration made before study start and being identical to the Clinical Trial registration and this detailed protocol in all important aspects. To increase transparency and facilitate comparisons, the SERA-protocol, written in English, is added to the supplementary materials (see supplement E).

The final treatment in this trial was concluded on the 14th of July 2024, the final follow-up interviews were concluded on the 21st of October 2024, thus the primary data collection for this trial has been finished excluding the final 12-month follow-up self-assessments which are still ongoing. Before inclusion of the last participant, the original version of this protocol was published on the 17th of April 2024 in the Open Science Framework (OSF) and can be found at osf.io/cs4bx/. The OSF-protocol registration was done in parallel to submission of an identical manuscript to a scientific journal (later rejecting it).

Data analysis will start in February 2025

The trial will follow the guidelines of Good Clinical Practice adapted for psychological treatment. Findings of the trial will be disseminated through peer-reviewed journals and scientific conferences. A slightly shorter version of this study protocol has also been peer reviewed by the Swedish Research Council before granting the project funding (grant number 2016–01961). The study will be reported in accordance with the Consolidated Standards of Reporting Trials-Artificial Intelligence extension (CONSORT-AI). The results will be published in peer-reviewed academic journals and disseminated to media and via the project website.

Using a predictive DST for ICBT raises ethical questions regarding the accuracy of predictions and who bears the responsibility for clinical decisions. There is a risk that the decision support makes incorrect predictions, just as all kinds of tests and human judgments can give incorrect indications. In the current study, the main risk is that patients who will have a bad treatment outcome are not indicated as “at risk patients” (red) by the DST and that extra efforts and adaptations of the treatment are therefore missing. If they are wrongly predicted to have a successful treatment outcome (i.e. as green), the therapist may give less support than they would otherwise have done.

To minimize the risk in cases where the DST gives incorrect indications, the predictions are not conveyed directly to patients but to the therapists who always make a clinical assessment of the DST information and have the final say on whether the treatment should be adjusted or not, together with their supervisor. This applies especially to the indication “green”, when the patient is expected to have a good outcome, and the recommendation is to spend slightly less time on the patient. It is emphasized here for the therapist that the DST cannot correctly assess all possible scenarios and that the therapist can therefore make a clinical assessment that more time should still be spent on the patient. The therapists are informed about what the DST bases its predictions on to be able to relate critically to them. In the end, the clinical judgment of the therapists decides, with the support of the supervisors. The only exception is when the decision support indicates “dark red”, when the therapist must carry out a telephone assessment with the patient even if the therapist considers it unnecessary. Since this always means more care, it is not considered a risk for the patient.

In general, there may be risks in healthcare using ML methods without properly exploring how this use works in a clinical setting and what shortcomings and opportunities it has. As the technical possibilities and computing power increase, studies such as the current one become important examples of how these possibilities can be implemented and evaluated in practice. Another overall advantage is that it can help determine how useful adaptive treatment strategies can be in general and provide additional knowledge about the degree of prediction accuracy needed for a predictive decision support to be clinically useful.

At publication the code for managing and analysing the data will be made available, after possible require anonymising steps have been taken to ensure compliance with ethical approvals.

As described earlier neither therapists nor patients will receive detailed information about differences in the treatment conditions between which they are randomised. The purpose of this is to prevent expectation effects for the therapists and placebo/nocebo effects for the patients. This means that the research subjects do not enter the study with full information about what each condition contains, which sometimes could mean a risk. In the current case, we assess that the clinical risk (for the patients) of not describing this part of the study in detail is very small, as all other information about what internet treatment entails and has advantages and disadvantages is clear and the overall information given about the difference between the study conditions is accurate, although not detailed. We do not consider that there is any risk that the therapists will suffer from this blinding.

In all psychological treatment there is a small risk of negative experiences in the form of psychological discomfort and that some patients do not improve or even deteriorate in their condition. There is nothing in this project that can be judged to increase the basic risk as the therapeutic methods used, and their expected mechanisms, are the same as in regular care. In addition, the frequent measurement of well-being and suicide risk included in ICBT means that the patients are more properly monitored than is customary in usual psychiatric care for these patient groups.

The following are the supplementary data related to this article.

Supplement A SOPHIA: general therapist ICBT manual Supplement A

Supplement B SOPHIA: Manual Decision Support Tool (DST) Supplement B

Supplement C Interview Guide - Dark Red SOPHIA Supplement C

Supplement D Therapist and patient questionnaires Supplement D

Supplement E SERA study protocol Supplement E

## CRediT authorship contribution statement

Pontus Bjurner (PB), Nils Hentati Isacsson (NHI), Fehmi Ben Abdesslem (FBA), Magnus Boman (MB) Erik Forsell (EF) and Viktor Kaldo (VK) have all contributed to the conception and design of the study protocol. PB and VK have been mainly responsible for drafting and writing the protocol, with contributions and critical reviewing of important intellectual content from NHI, FBA, MB, and EF. All authors have been involved in the final approval of the version to be published, and all agree to be accountable for all aspects of the protocol.

## Declaration of Generative AI and AI-assisted technologies in the writing process

During the preparation of this work the author(s) used Google Translate and ChatGPT in order to translate the following Supplementary materials from Swedish to English: *A. General therapist manual, B. Manual Decision Support Tool (DST), C. Interview Guide - Dark Red,* and *D. Therapist and patient questionnaires*. After using this tool/service, the author(s) reviewed and edited the content as needed and take(s) full responsibility for the content of the publication.

## Funding statement

This work was supported by 10.13039/501100004359Swedish Research Council (VR; 2016-01961), The Erling Persson Foundation (grant number not applicable), ALF Medicine (FoUI-987214, FoUI-962599, SLL20170708, 20180429), the Bror Gadelius memory foundation (129900321123 and 129900457224), KI Foundations (2018-02158), the L.J. Boëthius foundation (grant number not applicable), Psychiatry foundation (grant number not applicable) and KID Funding at KI (2018-00989).

Trial sponsor is Karolinska Institutet, 171 77 Stockholm, Sweden.

## Declaration of competing interest

The authors declare that they have no known competing financial interests or personal relationships that could have appeared to influence the work reported in this paper.

## Data Availability

Not applicable for a study protocol, this manuscript does not report data generation or analysis.
