# Supplementary material for: Study protocol for a triple-blind randomised controlled trial evaluating a machine learning-based predictive clinical decision support tool for internet-delivered cognitive behaviour therapy (ICBT) for depression and anxiety
Source: Internet Interv. 2025 Mar 3;40:100816. doi: 10.1016/j.invent.2025.100816 (PMC11925161; doi:10.1016/j.invent.2025.100816)
Supplement: Supplement A — SOPHIA: general therapist ICBT manual [file mmc1.pdf]

A.SOPHIA: general therapist ICBT manual

Version 2.3

Contents

ROUTINE – START OF TREATMENT FOR NEW PATIENT 2
Write your welcome message to the patient 2
ROUTINE – THERAPIST DAY 3
Managing patients on your therapist days 3
ROUTINE – JOURNAL NOTES 4
Instructions for notes in the star for what is to be documented 5
TIME LOGGING AND CRF 6
Automatic time logging of therapist time in the platform 6
Time logging and noting in CRF 7
What the symbols (flags) mean 7
Managing suicide risk 7
Inactivity 8
Close and note in the flag 9
Inactivity - despite the patient sending messages 9
7 days left of treatment 9
END OF TREATMENT 10
Therapy summary 10
CRF check and completion 10
Conclusion 10
ROUTINE – SPECIAL CASES 11
Patient wants to terminate prematurely 11
Extension of treatment time 11
Change of treatment 11
ROUTINE – ABSENCE/LEAVE 11
If you as a therapist have a leave lasting more than a week 11
GENERAL ADVICE FOR INTERNET THERAPISTS 11
General about the treatment work and providing support 11
General about feedback on module responses and messages 11
Content of feedback on module responses and messages 12
Therapist behavior in internet therapy (advice from the course videos) 13
ROUTINE – CHILDREN AS RELATIVES 13
Noticing children as relatives 13
Report of concern to Social Services 14

ROUTINE – START OF TREATMENT FOR NEW PATIENT

- The SOPHIA project coordinator initiates new patients for you in the platform. You will receive notification via email and/or SMS when you have been assigned a new patient.
- Log in to https://p2.internetpsykiatri.se/fou/admin.
- Click on "Participants" in the left menu, at the bottom under Select treatment choose "All treatments". Under the tab "My participants" you will see your patients and any flags.
- Once you have found your new patient, click on for the respective patient (You are now entering the patient view and see the platform as the patient sees it).
- Then click on Participant Editing in the left menu (this cannot be seen by the patient, you will now enter the therapist view, which you also access by clicking on next to the patient).
- Under the tab "Participant Information" read the assessment note from the journal in the "Notes" field to get a general overview of the patient.

Write your welcome message to the patient

- When you have been assigned a new patient and are about to send your own welcome message or respond to the patient for the first time:
- Click on "Therapist Contact" in the left menu and then select "Conversation View" (to see both messages from you and the patient).
- Read the general welcome message sent by the SOPHIA project coordinator.
- Write your own welcome message to the patient where you introduce yourself, mention that you will be in contact in the coming weeks, and reassure the patient that they are welcome to reach out with any questions. Refer to the SOPHIA standard texts appendix for an example template.
- The first time you write your welcome message, create your own template for it, e.g., in Word.
- Paste the text into the Message box, type e.g. "Welcome from your therapist" in the subject box, double-check that you have written the correct patient name, etc.

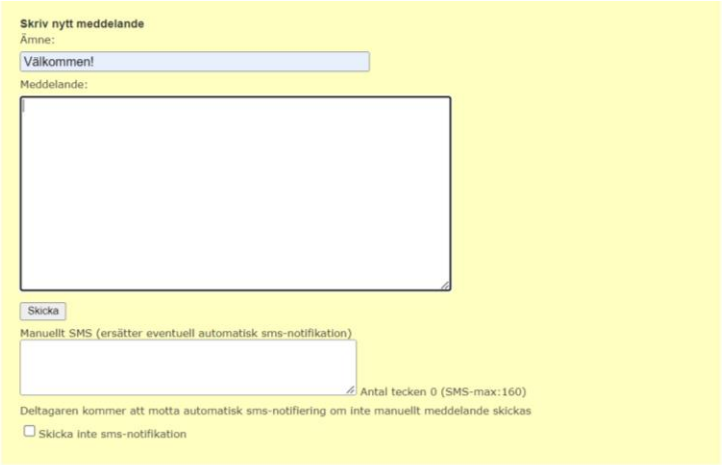

- Click "Send". (the patient will now receive an automatic SMS: "You have a new message on the website")

- Then leave this view by clicking on "To therapist interface" at the top and select "save" to log the therapist time you have just spent, which will automatically be displayed in the box. If necessary, you can edit the time (e.g., if you took a short break but remained logged in) indicated in the box or (in very rare cases) click on "No time" if you are not logging any therapist time. See separate instructions further down in the paragraph on Time Logging in the platform.
- If you have already received a message or module response from a new patient, you can respond to it according to the instructions under ROUTINE – THERAPIST DAY.

ROUTINE – THERAPIST DAY

Managing patients on your therapist days

- You log into the platform and manage your patients primarily on your three therapist days, which you plan yourself, according to the following rules:
    - At least one day between each therapist day.
    - The first therapist day is Sunday through Tuesday (preferably Monday).
    - The second therapist day is Tuesday through Thursday (preferably Wednesday).
    - The third therapist day is Thursday through Saturday (preferably Friday).
- No suicide risk assessments are made by us on weekends. For example, if one of your five patients has a suicide flag, the other four can be managed on the weekend, but the one with the flag will be addressed on Monday.
- If you have the opportunity and desire, you can also respond to messages from your patients on days other than your scheduled therapist days.
- Your therapist days can vary from week to week, meaning they do not have to be the same therapist days every week.
- On your therapist day, log in to P2 and click on Participants in the left menu, scroll down under Select treatment, and choose All treatments. Under the My participants tab, you will find all your patients.
- Here you can see which patients have flags and need to be addressed. If a patient has no flag, you can wait until the next therapist day (EXCEPTION: if you have arranged with the patient to contact them on that day).
- The most common flags are: or for unanswered messages from the patient or for inactivity. (Descriptions of all flags/symbols are found later in the cheat sheet).
- First, address patients with or flags via .
- Click on Therapist Contact in the left menu, then click on Conversation View to see new messages.
- Refer below for guidance on how to write messages under General Advice for Internet Therapists.
- Then follow the preparation routine:
    - Read the patient's messages/module responses.
    - Read what the patient has written in the current worksheets in the right menu.

- If necessary, read previous messages from the patient.
  - Then click on Participant Editing in the left menu.
  - Check the patient's login information in the Participant Information tab (last login, number of logins, amount of time) to get an idea of how active the patient seems to be.
  - If necessary, re-read the assessment note in the Notes field.
  - Look at the Graphs tab to see how the patient's well-being seems to be progressing. Is the patient improving, unchanged, deteriorating?
- After reading and preparing, start drafting your response message in a Word document (which you then delete without saving after sending it).
- Go back to Therapist Contact and Conversation View in the left menu.
- Reply to the patient's messages by clicking on the reply arrow next to the message and paste your edited response into the text box. Click "Send" when you are finished.
- If the patient has completed a module, click on Participant Editing and select the Treatment Information tab. Check the box for the next module and then click Save at the bottom.

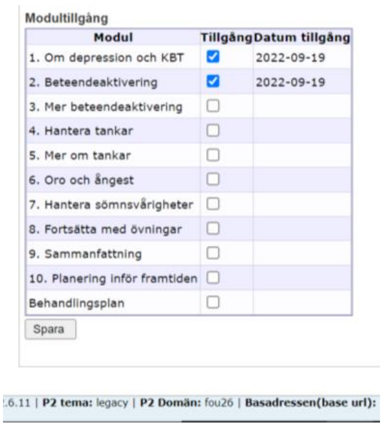

Modultillgång

Modul	Tillgång	Datum tillgång
1. Om depression och KBT	☑	2022-09-19
2. Beteendeaktivering	☑	2022-09-19
3. Mer beteendeaktivering	☐	
4. Hantera tankar	☐	
5. Mer om tankar	☐	
6. Oro och ångest	☐	
7. Hantera sömnsvårigheter	☐	
8. Fortsätta med övningar	☐	
9. Sammanfattning	☐	
10. Planering inför framtiden	☐	
Behandlingsplan	☐	

Spara

:6.11 | P2 tema: legacy | P2 Domän: fou26 | Basadressen(base url):

- If you think it would be beneficial to open the modules in a different order or open several at once, you must first bring it up in supervision.
- Finish by going to Participant Editing in the left menu and make a note in the "Temporary comment" box (the star field) according to the routines below ROUTINE – JOURNAL NOTE.
- It is IMPORTANT to ALWAYS, when you are finished with an individual patient, leave the patient view by clicking on To therapist interface and select Save to record therapist time. You can edit therapist time if, for example, you take a break during your work with the patient. See detailed instructions below under the heading Time Logging in the platform. (So do NOT click Log out when you have finished handling a patient).

ROUTINE – JOURNAL NOTE

- You write journal notes under the tab "Participant Information" in the box "Temporary Comment" (the star) according to the instructions in the table

below, (the notes are then transferred by the administrator to the Take Care (TC) journal system).

- You can access the patient either via + click on Participant Editing, or via to access the "Temporary Comment" box (the star) under the "Participant Information" tab.
- The following events should always be noted in the "Temporary Comment" box (the star) for journaling:
    a. Feedback on module responses.
    b. More extensive messages/responses in P2 to the patient, which may be considered part of the treatment, as well as messages due to Inactivity in P2. Thus not short responses to messages from the patient, like "Glad to hear that it's going well!". If you are unsure, always ask your supervisor. SMS should not be noted here, only in CRF (see separate CRF instructions).
    c. Phone calls to the patient containing any form of assessment or treatment.
- NOTE! It is important to always use the text format in the table's gray boxes and to only note what is specified here, so that our administrator can clearly see what needs to be transferred to the journal.
- NOTE! Do not write TC!

Instructions for notes in the star for what is to be documented

**Type of contact**	**Note in the star**
Response to the patient's module answers.	YYMMDD (date of the message)<br>Module response no. X /NN (your initials)<br>EXAMPLE:<br>220325 Module response no. 3 /PB
Message/response to the patient, or message due to inactivity	YYMMDD<br>Message /NN<br>Or<br>Message due to inactivity.<br>EXAMPLE:<br>220925 Message /PB<br>Or<br>220925 Message due to inactivity /PB
Phone contact	YYMMDD (date of the phone call)<br>Phone /PB

	(use the headings below)<br>Reason for contact<br>(select one of the options below)<br>Inactivity<br>Therapeutic conversation<br>Discussion about possible termination<br>Current<br>(brief overview of what was discussed in the call)<br>Assessment<br>(your assessment of the patient's well-being)<br>Action<br>(what action you/they took, e.g., some form of therapeutic intervention or providing the patient with information)<br>Planning<br>(what is planned going forward based on the above)<br><br>EXAMPLE:<br>220925 Phone /PB<br>Reason for contact<br>Inactivity<br>Current<br>Patient informs that they have been ill and unable to log into the platform<br>Assessment<br>Patient is motivated to continue treatment as planned.<br>Action<br>Discussion about how the patient can proceed with the next module.<br>Planning<br><br>Patient will submit the next module response by the end of this week.

- Bookings and notes in TC are made by the administrator with a maximum delay of 24 hours, (except for suicide assessments which are documented on the same day, see separate routine).
- Every time you have a treatment contact with a patient on the platform via message, a note in the "Temporary Comment" box (the star) should be made.

TIME LOGGING AND CRF

Automatic logging of therapist time in the platform

- Noting the time spent on each patient is a crucial part of the SOPHIA study, as it is a significant aspect of the evaluation process.

- This is done in two ways:
  - Through the platform's automatic time logging of how long you are logged in with a specific patient (via), including any manual adjustments you make to this time before leaving the patient.
  - By noting specific treatment actions you take in CRF in addition to the work done on the platform, such as calling the patient. What a CRF is and how to use it will be explained further below.
- In the platform's automatic time logging for therapist time (which appears when you leave the patient view by clicking To therapist interface), only the actual treatment work in the platform should be logged. This includes reading the patient's messages, module responses, and worksheets, writing and sending replies, as well as making journal notes in the star.
- If the time shown in the box is correct, click Save directly. If not, edit the time to make it accurate.
- If no time should be recorded, click No time.
- If too little time was saved, re-enter the patient via and add the correct number of minutes.
- If too much time was saved, make a note in CRF about it under that section.
- Time that should not be logged as therapist time via the platform's automatic time logging, and which you may manually need to deduct from the time suggested by the platform when leaving a patient, includes:
  - If you take a break while logged in with a specific patient that is unrelated to treatment, such as receiving a short phone call, getting distracted by something, remembering something urgent you need to do before continuing with patient work in P2, or briefly stepping away from the computer and forgetting to log out (which should be done for privacy reasons).
  - If a patient is called without the therapist being logged into the treatment at that time, and the therapist later logs into the patient to make a time note in CRF, THAT time should not be automatically logged as P2 time.
  - If you access a patient during supervision or to look at something or show something for a purpose unrelated to the patient's treatment, such as something related to research in the SOPHIA study.
  - Finding basic information about the treatment, such as reading treatment modules to learn about the treatment. However, quickly checking something in a module while writing a response to the patient should be logged as therapist time.
  - Supervision (group and individual).
  - Notes in CRF

Time logging and notes in CRF

A Case Report Form (CRF) is used in clinical research studies to gather everything important to understand the participant's journey through the study (as opposed to the journal, which only documents the care itself).

NOTE! Some activities are noted in multiple places, for example, if you call the patient, it should be noted both in the star journal and in CRF. The following are activities that SOPHIA therapists should always note and time-log in CRF (other CRF notes are made by the SOPHIA study coordinator, among others in the research group).

- Phone calls with the patient.
- Letters to the patient.
- Deviances such as: prolonged inactivity, the patient is away, the patient is ill, the patient has technical issues with P2 or login problems, the patient terminates prematurely, etc.

For further instructions, see the SOPHIA CRF Instructions A/B appendix.

What the symbols (flags) mean

Information about what each flag means will appear when you hover over the flag. Sometimes flags may appear duplicated, in which case you can close all extra flags and keep the one you need to handle. Flags are managed by accessing the patient via and clicking on the Flags tab.

= Message left today (after 00:00).

= Message left yesterday (before 00:00 today) or earlier.

= Scored 4-6 on MADRS-S suicide item (question 9 about zest for life).

= (usually) No message left for 7 days.

= (usually) No message left for 11 days.

= 7 days left until end of treatment.

= Patient's treatment time is concluded.

= Patient is in treatment but lacks a psychologist (NOTE! Only for coordinator).

= Measurement flag, hover over the flag to see which measurement it reminds of.

= Measurement delayed or missed, hover over the flag to see which measurement it pertains to. It could, for example, concern:

Patient has not filled out follow-up assessment 14 days after activation (i.e., 7 days after end of treatment).

Management of suicide risk

As a therapist, you never conduct any suicide risk interviews. That responsibility lies with supervisors and KAP.

- = the patient has scored 4-6 on the MADRS-S suicide item 9. NOTE! KAP has the main responsibility for this flag and monitors it daily.
- If patients write something that worries you in a message, you should immediately contact the supervisor or KAP.
- Patients included in treatment are managed by KAP based on the suicide assessment routine in the Suicide Assessment Guide.
- The clinical responsible psychologist (KAP) of the SOPHIA project has the main responsibility for monitoring and assessing suicide risk.
- The KAP of the SOPHIA project writes a note in the star indicating that management is ongoing, with the date and name of the person making the assessment.
- The KAP of the SOPHIA project closes the flag once it has been handled via Participant Information, Flags, and notes the action taken, e.g., "suicide interview conducted, low risk."
- The KAP of the SOPHIA project also makes a journal note in the star field (e.g., management, plan, or agreement).

Inactivity

Standard procedure (modifiable based on need in consultation with supervisor):

1. First flag = the patient has not written a message for 7 days. Send SMS, see templates in the SOPHIA standard texts appendix. SMS is sent via the External contact tab when you access the patient via or via Participant Editing if accessed via .
2. Second flag appears (usually after an additional 4 days) when the patient continues to be inactive. Send a platform message by accessing the patient via , see template in the SOPHIA standard texts appendix.
3. Third flag appears again after 2 days of inactivity. Call the patient, try repeatedly. The goal of the call is primarily to help the patient progress in treatment by making a concrete agreement with a deadline. Secondly, we should help the patient have a good conclusion if it is truly impossible to find a way forward. NOTE! Check with supervisor before deciding on closure.
    - After calling a patient - Always make a journal note in the "star". Remember to also note the phone call in the patient's CRF.

Calling

- When the -flag appears and you have already reminded the patient to contact you twice (first via SMS and then platform message), it's time to call.
- First, check previous activity. Many previous flags? When was the last login? Long intervals between messages? How is the treatment progressing? How many modules have been completed and how much time remains?

- Call the patient to discuss the next steps. Agree with the patient on how to proceed: Primary focus on continued treatment (possibly with deadlines for module submission or making a plan to work at a slower pace) or secondarily closure (possibly a follow-up visit for referral). Discuss the matter with your supervisor.
- NOTE! If there are signs of increased suicide risk and you cannot reach the patient, discuss with your supervisor.
- Close the flag when it has been handled. The standard practice is to reopen it in 4 days if the issue persists. Optionally choose fewer days. Note in the star field if there is anything that should be recorded.

Sending a letter

- If the patient does not respond according to the agreement you made over the phone, or if you cannot reach the patient after several contact attempts by phone, a letter should be sent. NOTE! After several contact attempts, make a note in the Star that you have tried to reach the patient.
- Contact your supervisor or coordinator for letter management.
- The letter should indicate when the patient will be discharged if they do not respond. Assess whether the patient should be called for a follow-up video visit if they are discharged and include this information in the letter if necessary.
- Make a note in the Star indicating that you have tried to reach the patient without success and that a letter has been sent, as well as any deadline specified in the letter.
- Also send a platform message with the content of the sent letter.
- Close the flag with reopening on the agreed deadline.

Close and note in the flag

Remember to always close the flag when it has been handled via the Flags tab. Then go to the patient via , click on the Flags tab, access the current flag via the symbol and make a note in the "message sent" box, check the box to close the flag with this note, and click save.
Manually change to 2 days until reflagging, write "SMS sent" in the box, and click save.

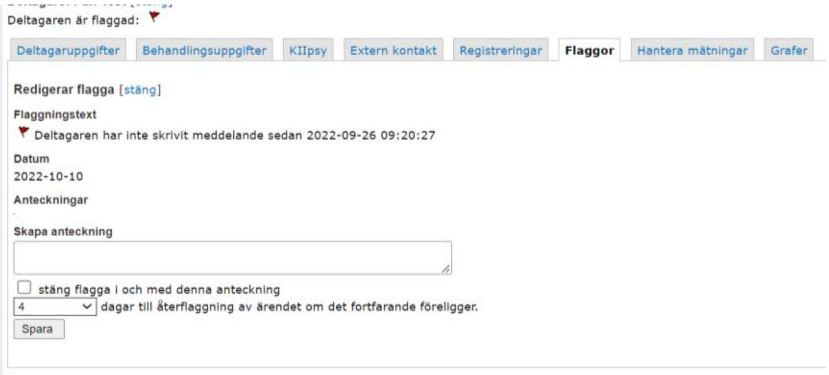

- Click on and note your action in the box, e.g., "Message/SMS sent". Choose the reflagging time, the standard is 4 days initially (after the platform

message) and 2 days (after the SMS, if the patient continues to be inactive). Save and close.
- If the patient sends a message after the flag appears but before you have contacted the patient, you can close the flag directly by clicking on quick close, if it hasn't been automatically closed.

Inactivity - despite the patient sending messages

- If the patient sends messages but does not submit module responses for 3 weeks, set a deadline for when the next module response should be submitted, and if the patient does not meet it, discuss alternatives with your supervisor. If the patient meets the deadline, continue to follow up with deadlines for the remaining treatment period.
- If the patient sends messages but does not progress in treatment, discuss solutions with your supervisor.

7 days left of treatment

- = 7 days left until the end of treatment.
- Send a message stating that there are 7 days left. Use the standard message found in the SOPHIA standard texts appendix.
- Send a reminder SMS via External contact. You will find reminder SMS in the SOPHIA standard texts appendix.
- Simultaneously with the 7-day flag, the follow-up assessment is activated, which is important for research and follow-up. When the patient logs in to read the message, they will necessarily have to complete the follow-up assessment first.
- After sending the message + SMS, write in the star:
- "230125 7-day sent /PB".
- In connection with sending the message, the last module in the patient's treatment should always be opened regardless of how far the patient has progressed otherwise. NOTE! Open only module 10; any other potentially unopened modules will be automatically opened after completion.
- The patient will receive a follow-up call (phone/video). Someone from the research team will contact the patient to schedule the follow-up call.
- Adapt the 7-day message you send to the patient based on your assessment.
- NOTE – keep the -flag open until you see that the patient has completed the follow-up assessment and responded to your 7-day message.
- Close the flag when the patient has completed the follow-up assessment via Flags.

END OF TREATMENT

The day after the last day of treatment, the stop flag appears:

STOP = Patient's treatment time is completed, which means that the therapy summary should be written, a new reminder SMS about the follow-up assessment may also need to be sent, and you must carefully go through that

you have made all the notes that need to be made in the patient's CRF (and check that these are filled in correctly).

Follow the steps below for therapy summary, CRF check and completion, and reminder about the follow-up assessment:

Therapy Summary

- Follow the template in the SOPHIA Treatment Summary Guide.
- Access the patient via and navigate to the Participant Information tab. Paste the summary into the Temporary Comments box with the heading: 221011 Summary /NN (your initials).

CRF Check and Completion

Ensure that the CRF for treatment interventions is correctly filled out and that you have completed the CRF for follow-up notes.

- Note the duration of treatment time automatically logged by P2 for the patient via and the Participant Information tab. Review this time and make an assessment whether it, along with the time logged for various phone calls etc. in the CRF, collectively represents a reasonable estimation of the time you have spent. This should be confirmed in a specific section of the CRF, where suggestions for correcting logged time should also be made if you discover any significant discrepancies that have been missed in previous corrections when logging out from a patient.

Reminder for Follow-up Measurement.

- NOTE! Send an SMS to the patient if the follow-up measurement has not been completed. This is crucial for quality monitoring and research purposes! Refer to the SOPHIA standard texts appendix for the message template.
- Once you have finished writing your treatment summary and pasted it into the "star" field, the SOPHIA project coordinator will take over the task of chasing any outstanding follow-up measurements.
- If the -flag appears for an undone measurement, make a final effort to obtain the follow-up measurement through an SMS reminder (but two reminders in total are sufficient). Therefore, the patient will receive two SMS messages if you follow the reminder routine for the -flag and -flag.
- When all closing procedures are completed, click yourself off as the therapist via under the Participant Information tab. At this point, the SOPHIA project coordinator will take over responsibility.

ROUTINE - SPECIAL CASES

Patient wants to terminate treatment prematurely

Call the patient

- Investigate the reason. Are there any misunderstandings that can be clarified? Is it related to performance anxiety? Can you motivate the patient to give it a few more weeks?
- If the patient feels that the program does not address their issues, inquire about what the patient wants help with and consider the possibility of

switching treatments (if the patient's issues/diagnosis fall within the scope of the SOPHIA project). Before any potential switch, always consult with your supervisor first before making a decision.

- If the patient decides to continue with the treatment, proceed as usual and make a note in the Star field.
- If the patient decides to terminate the current treatment, hand over to the SOPHIA coordinator for booking a follow-up appointment.
- NOTE - In case of elevated suicide risk or significant care needs, hand over to your supervisor.

Extension of Treatment Duration

Decision

- The decision to extend the treatment duration by a maximum of 2 weeks for an individual patient can only be made by the supervisor. Extensions are very rare and only granted for very specific reasons.

Change of Treatment

If the Patient Was Started on the Wrong Treatment Initially:

- Notify the coordinator, who will restart the patient on the correct treatment.

If the Therapist and/or Patient Contemplate Changing Treatment During the Course of Therapy:

- First, discuss the matter with the supervisor regarding the switch. Usually, it's advisable to have a discussion with the patient over the phone before making the switch. If necessary, such as when a new diagnosis needs to be made, the patient should be called in for a new assessment session with a psychologist.

Routine - Absence/Vacation

If you, as a therapist, have a vacation lasting more than a week:

- Check with your supervisor and send an email to the coordinator at least one week in advance stating the days you plan to be away.

General Advice for Internet Therapists

Regarding treatment work and providing support:

- The treatments are self-help treatments with psychologist support, meaning that the treatment content largely consists of modules with their worksheets. The psychologist acts as a support to guide the patient in treatment and assist when needed.

- Keep in mind that the need for support varies greatly at different stages of treatment and from person to person.

General Feedback on Module Responses and Messages

- Before you begin working on the module response or message, remind yourself of what the patient has been working on (by reading worksheets and module responses) and assess how they are doing through the preparation routine above.
- In providing feedback, the general approach is to highlight, reinforce, and commend what the patient has done and the steps they have taken in treatment.
- Feedback doesn't need to address everything the patient has written or done in the module; the most important thing is to assess whether the patient has grasped and internalized the content, and to help them progress in treatment.
- However, it's helpful to have a small section in the feedback that clearly addresses something the patient has written about in a response to a question or in a worksheet. This increases the likelihood that the patient feels seen and acknowledged because you show that you've noticed what they've written.
    - A good method for briefly reflecting/summarizing what the patient describes is to handle several difficult, but not treatment-related issues that the patient brings up with a summary reflection (where you can choose to highlight one of those issues): "I can really understand that you're struggling with everything you describe. One thing that I particularly thought about..."
- Avoid asking questions or follow-up questions unless it's about something extra important, as it risks leading to "parallel treatment" and taking focus away from the core methods.
    - If there's something you want the patient to think about more, questions can be reformulated, for example: "Please consider..." Or you can clarify that they are rhetorical, for example: "Questions to ask oneself might be: ..."
- In cases where the patient hasn't grasped the content or is having difficulty progressing in treatment, problem-solve and suggest strategies that can be used for the patient to move forward. If the difficulties concern module responses, assess whether the patient has internalized enough to move on to the next module or instead needs to work a bit more on the current one.
- Keep in mind that treatment time is limited. Remind the patient that it pays off in the long run to actively engage in treatment during the treatment period.
- The different treatments have different core methods. Keep the basic method(s) of treatment in mind and constantly return to this/these in contact with the patient. This makes it easier for the patient to maintain a thread in their work and focus on what is most important.
- For example, behavioral activation and cognitive restructuring for depression treatment, and exposure/behavior experiments in anxiety treatments.

Feedback on Module Responses and Messages

The structure and content of a module response or message may vary, but common elements to address, roughly in this order, include:

- Encouragement for how the patient has worked on the treatment.
- Validation of what has been challenging in the treatment process or how the patient is feeling.
- Positive feedback on what the patient has understood and accomplished. It's often helpful to summarize this in a paragraph rather than repeatedly going through each part separately. Also, explain why it's beneficial that the patient has understood or done this particular thing and how they may use it in the future.
- Feedback on the content of worksheets or something the patient has written in the module response. Highlight something you believe is especially important for the patient to continue working on or an insight that may be useful to reinforce.
- Correction if there is something the patient has misunderstood. To maintain a friendly tone, it's helpful to link the correction to something the patient has actually understood. Also, remind them of the rationale for the method when correcting. Refer primarily to the modules instead of writing your own lengthy explanations.
- Something the patient may benefit from focusing on in the next module or in general. Is there something in the core method the patient should focus more on?
- Connect something the patient or you have written to the next module or something that will come later in treatment. This way, you can convey hope and clarify how the patient should continue working and what comes next.
- Closing words indicating that the next module is open and the patient is welcome to reach out with questions and anything else.

Note that a message rarely includes all these parts; a condensed version of all the points above is that a message should include: validation, problem-solving, and forward direction.

Therapist Behavior in Internet Treatment (Advice from the Course Videos)

- Be a therapist (not a friend) in your interaction with the patient.
- Start and end your messages "formally."
- Be personal but cautious with jokes. Jokes can become avoidance or make the patient afraid to be serious.
- Validate and confirm.

- Example of validation: "I notice from what you're writing that you're really struggling right now. It sounds incredibly tough to go through so much in such a short time."
- Confirmation is not the same as praise. Example of confirmation: "I'm glad to see that you've really committed to exposure! It also seems to have had a positive effect so far, don't you think?" Be specific instead of generally encouraging.
- Explain misunderstandings (if important). A correction is often punitive.
- Try to reinforce while explaining when it's important. Reinforce desired behaviors, extinguish or address unwanted behaviors. This includes not at all, or very briefly, responding/commenting on long descriptions/stories that patients make but that are not clearly related to treatment.
- Use summaries. Clarify what the patient is doing that is effective.
- End by describing the next step and giving encouragement.

ROUTINE - CHILDREN AS RELATIVES

Attention to children as relatives

- As therapists, we have a responsibility to be aware when minor children of our patients need information, advice, or support. To address this, the patient receives information about the document "To You with Children in Your Environment" in their first message from us. This document provides advice on how the patient can talk to their children about their difficulties. The patient is also encouraged to ask their therapist about this topic if needed. The therapist can provide advice through messages on the platform or phone calls if deemed appropriate. We do not offer reception visits focusing on the child's perspective.
- If the patient does not come with a specific question about the children, it may still be appropriate for the therapist to address issues related to the children. This is done based on the therapist's own assessment of the situation. Discussions on this topic can be initiated if, for example, the patient mentions being easily irritated with their children or if it becomes apparent in any other way that the patient's well-being is affecting the children.
- If support is needed, please consult the child advocate (via the supervisor).

Report to Social Services

If there is concern for a patient's child (or any other reportable event) during assessment or treatment, an immediate report should be made to social services.

It is possible to call social services in the patient's municipality in advance to consult and, if necessary, receive further instructions (you can discuss the case without providing identifying information if you first want assistance in determining whether a report should be made or not).

Therapists consult with supervisors before making a report, but legislation always determines how each individual acts. Everyone has a responsibility to follow the law.

The patient should be informed about the report before or at the time it is made. If this is not possible, the patient should be informed of the action afterwards. There are cases where the guardian is not informed that a report is being made/has been made, such as when the guardian has subjected the child to violence. In such cases, it is advisable to call Social Services in advance for advice. Assess whether the patient should be offered an appointment at the clinic to discuss the report and the situation further. In some cases, you may have already met with the patient and discussed the report or had a discussion over the phone. Seek assistance from managers/head physicians/child advocates in your assessment regarding the need for a follow-up appointment if necessary.
