# Supplementary material for: Study protocol for a triple-blind randomised controlled trial evaluating a machine learning-based predictive clinical decision support tool for internet-delivered cognitive behaviour therapy (ICBT) for depression and anxiety
Source: Internet Interv. 2025 Mar 3;40:100816. doi: 10.1016/j.invent.2025.100816 (PMC11925161; doi:10.1016/j.invent.2025.100816)
Supplement: Supplement B — SOPHIA: Manual Decision Support Tool (DST) [file mmc2.pdf]

B. SOPHIA: Manual Decision Support Tool (DST)

Purpose of the decision support tool

The decision support tool is a guide on how clinicians should act with different patients and make clinical decisions. The decision support tool consists of two parts:

1. The technical interface that shows information about how things are going and predictions for how the treatment is likely to progress for a particular patient. The predictions concern symptom assessments and the level of activity in the treatment. Here, a summary recommendation in the form of a color signal is provided to you as the clinician.
2. This quick guide that describes in detail how to interpret and act on the information and recommendations provided by the decision support tool.

Background to the decision support tool

Several previous studies have shown that clinicians can benefit from support in assessing and monitoring how a patient's treatment is progressing and what the final outcome will be. It is natural for clinicians to focus on helping the patient here and now, and as clinicians, they may have an optimistic view of the treatment outcome and convey hope to the patient. This, among other factors, can make clinicians somewhat blind to when treatment is stagnant or not progressing in the right direction. Many studies have shown that when clinicians are helped to pay attention, especially to treatments that are at risk of achieving inadequate or even negative results, the final outcome improves because adjustments can be made in time.

How does the decision support tool work?

A central part of the decision support tool is, as mentioned above, the prediction made regarding the final treatment outcome the patient will receive. The decision support tool helps the clinician determine which patients need extra help, which can be expected to manage with a standard level of assistance, and which seem to be achieving a good outcome and may manage with fewer and shorter interventions from the clinician. The quick guide then provides closer guidance on what the clinician should do. The hope is that the clinician's time is used better and more tailored to each patient's needs, and that overall treatment effects are improved.

In this study, we are testing predictions made using machine learning (ML). Since internet-mediated treatment collects a large amount of structured data, there is a good basis for using ML methods. The purpose of this is to provide clinicians with automated predictions with high precision.

Here is a brief description of what ML is and what it does in this project. Wikipedia defines ML as follows:

> *"Machine learning is a field within artificial intelligence concerning methods for 'training' computers with data to detect and 'learn' rules to solve a task, without explicitly programming the computers with rules for that task. The field overlaps with statistics, computer vision, and pattern recognition."*

For this study, machine learning (ML) has been used to train on clinical data for approximately 6000 patients who have undergone the same treatment program as the one we are using. With ML, complex patterns are identified in a very large amount of data, including relationships between these patterns and the patient's treatment outcome. With support from the training, various ML methods can make predictions when given treatment data from a new patient. The data that the machine has been trained on and analyzes for new patients include:

- Patient's gender, age, and any comorbidities.
- Date of treatment initiation.
- Symptom ratings for primary diagnosis + depression (sum of weekly ratings + pre- and post-assessment, selected items, and timing of assessments).
- Homework assignments (if the patient has filled out the homework report, number of reports sent, and number of characters).
- Messages (Number and length for patient and therapist).
- Worksheets (Number of characters and words).
- Logins (number, timing, number of days with and without login, longest period without login, total time logged in).
- Treatment credibility scale, sum week 2.

Different data have different importance for the predictions. Roughly speaking, we can say that on average, predictions are influenced by 55% by symptom ratings, 30% by homework assignments, and 15% by other variables.

The decision support tool's prediction of the patient's symptom-rated treatment outcome is then categorized as successful or unsuccessful treatment. The threshold for successful treatment is defined in two complementary ways:

1. The outcome falls below the remission threshold, i.e., the threshold for when the patient is considered ill. Patients who fall below this threshold are usually referred to as remitters.

2. The outcome corresponds to a reduction in symptom ratings from before to after treatment of 50% or more. Patients who reduce their symptom ratings by 50% or more are usually referred to as responders. Using a percentage change is considered fair regardless of whether the patient starts with a very high rating or is relatively low. For example, it is probably equally difficult/good to go from 40 to 20 on MADRS-S as it is to go from 20 to 10.

Limitations of the decision support tool and your interaction with the decision support tool

The decision support tool provides predictions and advice on how therapists should act. The prediction is a strong signal, but it is not 100% certain, so it is always the responsibility of the therapist and supervisor to make the final decision on what to do.

In addition to the information from the decision support tool, decisions in treatment are guided by a general precautionary principle, to err on the side of giving too much treatment rather than too little. For example, when there is a change in colors in the recommendation, if a patient who was previously classified as red later becomes green, the therapist sticks to the red adapted plan for a period. At the same time, in the reverse scenario where a green patient becomes red, the therapist immediately abandons the green plan and starts to assess what actions the patient needs. In other words, it's better to adapt and provide extra measures a little too much.

It is important to consider that although many factors are taken into account in the decision support tool's prediction, it knows nothing about other factors that may affect the patient. This is often referred to as the "Broken leg" scenario, where a very rare event with significant impact occurs. It is impossible for the decision support tool to account for and is therefore something that therapists must consider in their clinical assessment. The clinical assessment should be based on a comprehensive picture that takes information from the decision support tool + other facts that the therapist has about the patient and treatment.

A clear strength of the decision support tool compared to humans assessing is that it does not get stuck on any particular factor or in any particular "causal idea" and thus forgets to consider others, nor is it impulsive or easily influenced.

General description of the graphs

Recommendation

This color signal is the primary guidance for you as a therapist. The color signal continuously categorizes the patient into one of four different colors. The colors are based on the decision support tool's prediction of the patient's symptom rating at the end of treatment and constitute the overall recommendation to you as a therapist. Detailed

guidance for the different color categories is provided further down in the section Guidance based on the decision support tool's recommendation and graphs. General description of the color categories:

Green – the patient is likely to do well = the symptom rating is likely to fall below the threshold for successful treatment. The therapist should consider spending less time on the patient.

Yellow – the prediction is not confident enough to give a green or red recommendation. The therapist works according to the usual setup in the Therapist Guide. After week 6, the decision support tool will no longer show yellow.

Light red – there is a risk that the patient's treatment will fail, i.e., that the symptom level will not fall below the threshold for successful treatment. The therapist is recommended to take certain actions and be more vigilant about further developments.

Dark red– there is a significant risk that the treatment will fail. This means that the therapist must take certain actions and is recommended to consider more than that.

Further down under each color category, detailed descriptions are provided of how the therapist should, or in several cases, MUST act. The extent to which you should follow the instructions based on the color signal varies; some actions are essentially mandatory, while others are more optional based on your own clinical judgment. If you feel unsure, always consult your supervisor. Generally, you should always consider your own assessment of the patient, and if something seems wrong or strange, you should discuss it with your supervisor. You must not deviate from the decision support tool and the guide without consulting your supervisor.

Actual values and predicted outcome with confidence intervals

This graph shows the actual weekly ratings that the patient has responded to so far, as well as the decision support tool's latest, best prediction of the outcome. The prediction is displayed with two confidence intervals (90% and 50%) indicating how confident the prediction is. If the patient has missed filling in a weekly rating, you will see a red cross between the other weekly ratings. The graph also includes a dashed line indicating the threshold for "successful treatment," meaning the threshold for remission and/or response.

This graph, along with the graphs Activity Index and Historical Predictions of Outcome per Week, are intended to provide the therapist with background information to analyze the patient's situation and treatment. They complement the color signal in the Recommendation and do not provide specific guidance on how to act.

Activity Index

This graph shows with the gray horizontal bar how active the patient has been since the start of treatment, compared to how active patients usually are in the week the patient is currently in. The gray area shows from 0 to 100% how active the patient has been in the current week. The black dot, with confidence intervals at 50% and 90% shown in two shades of blue, is a prediction of the patient's total activity throughout the treatment at completion, also in relation to how active patients have historically been.
Historical predictions of outcome per week
This graph shows how the predicted outcome has developed week by week, with confidence intervals (50% and 90%).

The daily work with decision support tool

When you log in to P2 to start a work session, do the following:

1. Right-click at the bottom right of the page on the link named "Services" and select the option "Open link in new tab":

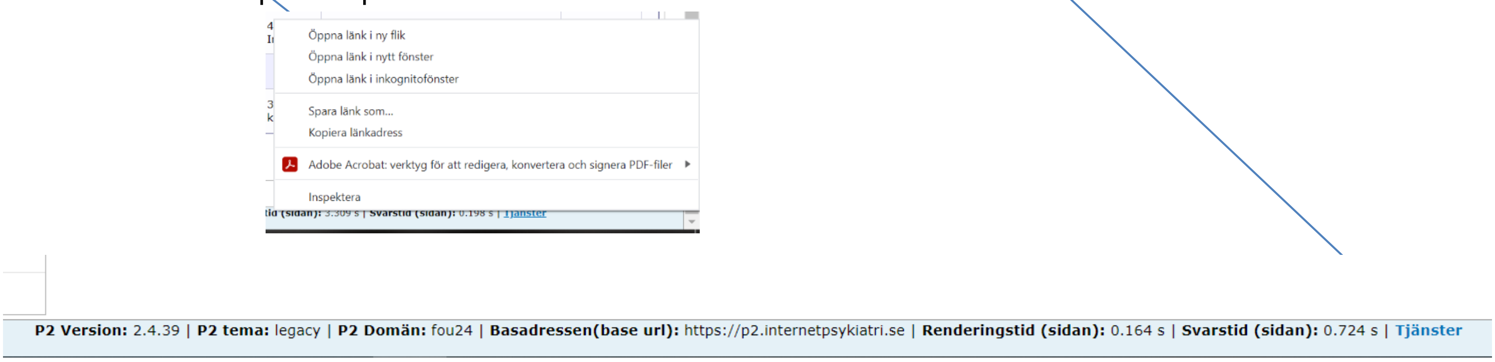

2. Go to the new tab/page and click on: **To DST**

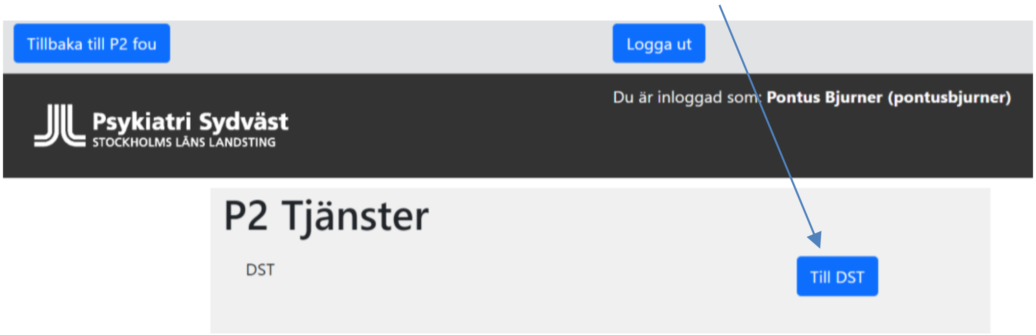

3. Use/look at the decision support tool **every time you work with a patient in P2.** Remind yourself if there is already a plan for adapting the treatment from the previous week and how it relates to the current color category. Look at the patient's information in the decision support tool and here in the guide to determine how to proceed with the patient.

4. **NOTE! Important EXCEPTION!** Yellow, green, and light red patients who notify that they want to terminate/discontinue treatment should be considered dark red and handled according to the instructions below.
5. At the end of the workday, or during longer breaks, log out and close the decision support tool.

Guidance based on the decision support tool's recommendation

General principles regarding decision support tool and treatment adaptation

The SOPHIA study is based on adapting treatment using decision support tool for patients who need it, thereby preventing treatment failures and reallocating time to patients with the greatest need and where it can make the most difference. Patients receiving adapted treatment and extra support should indeed receive more time and be treated more individually – especially in terms of the format of the contact, but also, if necessary, with other interventions such as problem-solving, extra modules, or spending more time adapting existing techniques based on a behavioral analysis of the patient's situation/problem. At the same time, we should not provide extra support unnecessarily and therefore distinguish between light red and dark red patients. Both categories likely require adaptation, but for the light red ones, we start with a less time-consuming assessment/analysis, while for the dark red ones, we delve into analysis, support, and adaptation more quickly and extensively.

Yellow

General guideline

This recommendation corresponds to how you should work with the patient based on the general guide. That is, you should not do anything extra, but you should also not minimize your effort. You follow the general guidelines in the general guide for writing messages, managing any flags for inactivity, suicide risk, etc. You spend an average normal amount of therapist time (=15 min/week) and should have a normal level of vigilance, continuously assessing how the treatment is progressing, and guiding the patient forward within the normal framework. Yellow indicates that we do not know with sufficient certainty how it will go for this patient and therefore do not increase or decrease the intensity of the treatment work. If the therapist wishes to deviate from the guide, it must first be discussed in supervision with the supervisor.

Until week 1, all patients are yellow regardless of the predicted final value. From week 2 to week 5, the yellow recommendation is gradually phased out (see table), and from week 6, yellow disappears completely, and patients are only categorized as green, light red, or dark red. The reason for this is that the general safety in prediction is initially lower and then

increases, so we want to be a bit cautious about how we categorize and adapt treatment in the first few weeks before becoming clearer from week 6.

Week	The proportion of yellow patients
1	100%
2	70%
3	50%
4	25%
5	10%
6	0%

Special scenarios for yellow patients and how to act:

- If a yellow patient has previously been light or dark red, you should stick to the previous planning and the actions that have been agreed upon.
- If a yellow patient has previously been green, you should immediately stop minimizing the amount of time you spend on the patient and follow the guidelines outlined for yellow patients.
- If a yellow patient who was previously light or dark red continues to be yellow after one week, an assessment should be made in supervision about when and if it is time to scale down the action plan.

Green

General guideline:

A green recommendation means that it is highly likely that the patient will do well, and therefore, you should focus on not doing more than necessary in the treatment. One purpose of the decision support tool is to help you better distribute your time based on each patient's needs. For green patients, it is primarily about limiting your working hours so that you can spend more time on patients with greater needs, i.e., light and dark red patients. For patients categorized as green by the decision support tool, it is crucial that they average no more than 10 minutes of therapist time per module. Some green patients and modules may take slightly longer, but this should be compensated by other green patients and modules progressing more quickly.

Rules for green patients:

- Maximum of 10 minutes of therapist time per module on average throughout the patient's treatment. In exceptional cases, more time may be spent on an individual patient, but this should be compensated by shorter times for other patients or later modules for the specific patient.

- Never introduce new material or new methods.
- Never start early with upcoming content by writing in messages how to, for example, work with mindfulness; instead, refer to it coming in module X.
- Avoid referring to other help during the treatment - see above about what to do if the patient has many other things they want to address.
- Do not spend more than 10 minutes per week chasing a patient classified as green even if they are not progressing in treatment. If you reach the patient after a long time/many attempts, continue "as usual" and stick to 10 minutes per module.
- Be careful to stick to the main diagnosis of the treatment. This applies primarily to panic disorder and social phobia. Depression is a broader diagnosis, so for these patients, it is okay to focus on other problem areas in the patient's life that likely affect depression.
- For panic disorder and social phobia, it is easy for a therapist to digress into topics that seem to affect the main diagnosis (e.g., stress management, pain, relationship problems, unemployment, conflicts, etc.), but in these treatments, we cannot and should not solve all of that. Instead, we focus on what the treatment is designed for, thereby giving the patient better conditions to address their situation (see below for specific tips on how to write). Of course, you can validate that the situation is difficult if it is, and provide contact information for, e.g., women's shelters, encourage contacting a job coach, or refer to discussing the problem at follow-up after treatment, but this can be done in a short sentence, and it is not where the focus should lie, i.e., as a therapist, you do not need to follow up and push for it. Keeping it brief about other issues is also a clear way to demonstrate which parts of the treatment to focus on - to socialize the patient into the treatment. Also, remember that the patient sought treatment for their main problem, and it is validating for you as a therapist to focus on that.
- If you want to deviate from these rules, you <u>must</u> discuss it with your supervisor first.
- Also, read through what extra support entails so you know what <u>not</u> to do with a green patient.

Special scenarios for green patients and how to act:

- If a green patient has previously been categorized as light or dark red, you should adhere to the previous planning and agreed-upon measures.
- If a green patient was previously categorized as yellow, you should immediately start minimizing the time you spend on the patient.
- If a green patient who was previously categorized as light or dark red continues to be green after a week, an assessment should be made in supervision about when it might be time to scale back the action plan.
- After week six, the yellow recommendation disappears completely, which means that a patient who has been yellow for several weeks becomes green. If you have a green patient following that pattern, it may be good to continue seeing that patient as more yellow than green and rather follow the yellow guidelines.

Help strategies to limit therapist time with green patients:

Patients who bring up many other problems:

- If normal socialization does not help, a feasible approach may be to actively request permission to focus on organizing the treatment's focus, for example: "You have brought up many things that I understand are affecting you very negatively - what you describe sounds very difficult, and I really understand that you do not want it that way in your life! At the same time, I don't see that we can solve everything in this treatment, so my suggestion is that we now focus on depression/panic disorder/social phobia, as it has proven to be the best way to get things in order. It is also true that your main problem affects a lot of other things, and therefore, there are all possibilities that some of what is problematic right now can resolve itself when it gets better. Then we can also arrange it so that if you want help with other things when we're done with the treatment, I can help you find a treatment where you can work on them. Would that work for you?"

Participants who communicate very frequently:

- Respond succinctly. If many (follow-up) messages are received, consider postponing your next response for a couple of days. Especially if the participant's messages are mostly social, this is a good strategy. For participants who are doing well, you don't need to work on the relationship by being extra social and responding to everything (that's what extra support is for!).
- If you decide to postpone the message(s) from a participant, send a brief reply to the participant stating something like "I will get back to you with a response on Friday." Then, do not read the participant's message until you plan to respond to it later.
- Carefully consider each follow-up question you ask. Does your question relate to the main problem or techniques/strategies/difficulties directly related to the treatment? If not, consider whether the question is really necessary. For participants who are doing well, you don't need to work on the relationship by being extra social and commenting on everything (however, it's great to do this with Extra Support!). Your own writing behavior affects the participant a lot - think about how you socialize the participant into the treatment.
- Sometimes, it may be necessary to explicitly state that you do not have the opportunity to respond to everything the participant brings up. In a polite way... (see example above).

- If you have needed to write at length to clarify something, remember to then move on by responding briefly and focusing on the main problem, even with long-winded participants.
- It is entirely acceptable to "hold the participant" (accommodate their anxiety!), validate, be kind/understanding, problem-solve around the current module/homework, clarify the material, and explain the methods in other words (but not to exceed 10 minutes per module).
- Feel free to mention that "It will come later, in module X" if the participant seems to need or request to work with a technique or method that comes later in the treatment.

Light Red

General Guideline

It is relatively certain that a light red patient is at risk of achieving a "failed" treatment outcome and therefore the treatment should be adapted. However, compared to a dark red patient, the risk of failure is lower, and therefore the assessment of problems and actions/adaptations is not as extensive and time-consuming as for dark red patients. Instead of directly calling the patient and conducting a full interview, you should start by activating an assessment form on the treatment platform and ask light red patients to log in and fill it out.

Assessment of Problems and Barriers Regarding Treatment

Activate the P2 form **SOPHIA Assessment Light Red** and send an SMS to the patient urging them to log in and fill out the form.
Example of SMS:

(NOTE! Adapt as needed)

"Hi (patient's first name),

Log in to the platform and answer a few questions that can help us adapt your treatment in the best way possible. Regards (your first name)"

If the patient has not responded within two days to the assessment form, send a new SMS reminding them to log in and fill it out by the next day, and mention that you will try to reach the patient from a hidden number thereafter. Make a reminder to yourself to call next week if the patient has not filled it out.

Call the patient the following week if the form is still not filled out. In the phone call, go through and fill out the form with the patient. EXCEPTION:

1. The patient has changed color. Then follow the guidelines for the new color.
2. If there are 2 weeks or less left of treatment, you should call the patient the day after the first SMS if the form is not filled out.

Assess based on the patient's responses in the assessment form (in combination with other information you have about the patient) what obstacles or problems you can see in relation to succeeding with the treatment. Assess if these are significant enough and let them form the basis for one or more adaptive measures.

Adaptations and Measures

If no significant problems have emerged, you can wait to take any actions, provided that your supervisor agrees with this assessment. However, make a note to yourself to actually take at least one action after another 1-2 weeks if the patient is still light red.

Keep in mind that the measures overall should be less time-consuming for light red compared to dark red patients. Refer to the "**Measures for adapting treatment for light and dark red patients**" section further down in the guide. There, prioritize measures marked with XXX. Check your proposal with the supervisor before sending it to the patient.

1. Activate the extra module "**Treatment Plan**" for the patient via and the "**Treatment Tasks**" tab, check the box for the module, and save.
2. Then, go to the patient via , click on the worksheet "**Treatment Plan**," enter a proposal for an action plan there, and save.
3. Next, send a message in P2 to the patient urging them to read the worksheet/action plan.

You can suggest changes to the plan directly in the worksheet "**Treatment Plan**" and then respond with a message about how they view the actions. If the patient does not respond to this message within two days, send an SMS stating that you will try to reach them from a hidden number and call the patient the following day. The focus of the telephone call is to determine and agree on an action plan.

Special Scenarios for Light Red Patients and How to Act

- If a light red patient has been green or yellow before, you should immediately follow the light red guideline.

- If a light red patient has been dark red before, you should stick to the dark red action plan. Even if the patient continues to be light red, the dark red action plan should be followed. If you feel that the action plan needs to be changed, it should always be checked with the supervisor first.
- If there are 2 weeks or less left of the treatment, try to quickly establish telephone contact with the patient and determine actions adapted to the remaining time of the treatment, i.e., actions that can have a quick effect and actions that the patient can continue to work on independently. Please refer to and partly base it on the content of Module 10 + **Measures for adapting treatment for light and dark red patients**, below.

Dark Red

*(**NOTE!** Yellow, green, and light red **patients who express a desire to terminate/discontinue treatment** should be considered dark red and handled according to the instructions here.)*

General Guideline

Dark red patients should be contacted as soon as possible to conduct an assessment/problem analysis and make adjustments and interventions based on that. We essentially want to do everything we can to prevent the patient from failing (within certain limits). The focus is still on the main diagnosis, but we may consider partially supporting the patient with other problems, especially if they appear to be a barrier to treatment.

Immediate Actions and Assessment of Problems and Barriers Regarding Treatment

You must immediately call the patient and conduct a structured telephone interview based on the document: **Dark Red SOPHIA Interview Guide**. Remember to follow **Mandatory Approaches and Interventions for Dark Red Patients** below.

It is important to establish contact with the patient quickly!

Often, dark red patients are difficult to reach, so there is a risk of having few weeks left to work with if the patient is not reached quickly. Therefore, spend extra time trying to quickly get the patient back on track. Remember to note all your attempts to reach the patient in **SOPHIA CRF DST** according to the instructions in the treatment guideline.

1. As soon as you see that the patient is categorized as dark red, try to reach the patient by phone (weekdays + daytime). If there is no answer, send the following message to the patient via P2:

   "Hi [patient's first name],

   I would like to schedule a check-in with you to hear how you feel about the treatment and if there's anything I can assist you with. [Something personal, e.g., It seems like it's been a bit challenging for you to get started with the treatment. /You've mentioned that ___ /something problematic/]. Therefore, I would like to give you a call so we can discuss how the treatment is going for you and how we can help you get the most out of it. I'll try to call you on [XXX day] at [XX o'clock] [within two days]. It's good to know that we almost always call from a "hidden number."

   If that time doesn't work for you, it would be great if you could send a message and suggest some times between [X and Y] on [X day, Y day, or Z day] when I can reach you [within the next three days].

   Looking forward to speaking with you!

   Best regards,

   [Your first name]"

2. If you haven't already reached the patient by phone, call at the agreed-upon time (i.e., within two days). If there's no answer, try again at least a couple more times during the same day. Be proactive in your attempts to reach the participant, and remember to note all your contact attempts and contacts in **SOPHIA CRF DST** according to the instructions in the treatment guideline. Call from both hidden and visible numbers (if possible) and at different times. For the study's sake, there is essentially no upper limit at this stage for how many times you reach out in different ways (social conventions and practical possibility set the limits). Also, send a text message at the end of the day if you haven't reached the person, with a personal message, approximately:

   "Hi! I tried to reach you by phone today but didn't succeed. I'll try again; please send me a message in the platform about when I can reach you! [Your name]".

3. Continue trying to reach the patient by phone several times over the next few days. At the end of each day, if there's still no contact, send a new text message with roughly the same content as above.

   If there's no response four days after categorization, send a letter according to the following template, preferably with some adjustments to make it more

personalized for the patient. The SOPHIA project will assist you practically in sending the letter; you just need to write the text and then contact the project coordinator.

"Hi [Patient's Name],

It's been a while since we last spoke, and I'm wondering how you're doing? When you signed up for this treatment, it was because of issues with depression/panic disorder/social phobia, and we would really like to support you with that. We've seen that this type of treatment can help many, and there's nothing that makes me believe you wouldn't benefit from it!

However, I understand that it can be really difficult to find time, energy, or understand how the treatment should proceed. That's why I'm here - to do whatever I can to make it possible for you to benefit from it! I have many suggestions on how I can help you get something out of this treatment (preferably as much as possible), and you probably have ideas yourself on how it could be changed to better suit you and your situation.

Many find it easier to talk on the phone than to send messages, and I'm happy to call you if you prefer it over sending messages in the platform. For example, we could have a scheduled phone call at regular intervals during the treatment when I call you, or we can come up with something else that suits you!

I've tried to reach you at phone number XXX-XXXXXXX but haven't succeeded. I'll try again on [X day], at [XX o'clock], so we can try to figure out how I can help you? If the number is incorrect, or if you prefer another time or day, please send me a message in the platform with a suggestion. You can also call the SOPHIA project coordinator at [phone number] if you have login issues or tell them when I can best reach you and on which phone number. Please note that we often call from a hidden number, in case you're accustomed to not answering such calls.

Warm regards,

[Your first name]"

4. Continue trying to reach the patient by phone and SMS after the letter has been sent. If the patient still hasn't responded after 8 days from categorization, send a summons for a telephone meeting.

5. When the patient responds, conduct an interview following the document "**Intervjuguide mörkröd SOPHIA**" with the aim of understanding how you can

assist the patient and to design a treatment plan. Activate the extra module "**Behandlingsplan**" and enter proposed action plans in the worksheet "**Behandlingsplan**". Then, send a message in P2 to the patient urging them to read the proposed actions/adaptations in the "Behandlingsplan" and to respond with confirmation or suggestions for changes to the plan.

6. If you're unable to conduct an interview when the patient responds (e.g., because the patient refuses to talk on the phone), seek supervision. The goal of the interview is to identify barriers to treatment and determine how you, as the therapist, can help. If there are actions that can be taken even before a problem assessment interview is conducted, it's unfortunate if these are delayed because the interview takes time to arrange.

Required approach and interventions for dark red patients:

A. Always conduct the telephone interview **"Interview Guide Dark Red SOPHIA"** as early as possible.
B. Act quickly even after the initial contact with the patient, as it's important that you provide prompt responses to what the patient sends and also promptly reach out if the patient is passive or dragging out the process.
C. Highlight early and clearly that together you can create a tailored treatment approach and provide a brief suggestion of what it might entail based on something that would likely be relevant for this particular patient.
D. As a therapist for a dark red patient, you should act more as a therapist than as a coach. The difference is that a coach primarily supports a patient through a self-help program based on the treatment material, while a therapist spends more time analyzing the patient's implementation problems and communicating with the patient about specific issues to explain, troubleshoot, support, and motivate.

Actions for adapting treatment for light and dark red patients

The following actions can be freely used for dark red patients, while there are more restrictions on which ones should be used for light red patients. The basic principle is that light red patients mainly receive actions that don't cost the therapist much time, while there is no predefined time limit for dark red patients. After each point, there is a brief guidance in [light red] to indicate how you as the therapist should approach if it's a light red patient.

1. Ensure that there is a customized Treatment Plan that includes the following actions agreed upon by the therapist and the patient. Ensure that the patient understands this.
   The Treatment Plan **should at least** include a concrete description of:

   a. What the patient should particularly focus on during treatment and when and how it should be done.
   b. If the patient should report on this to the therapist (e.g., every X days, with every homework assignment report, when accomplishing Y).

   [Important but often less detailed for light red. Less time is spent on drafting the treatment plan, and it is primarily based on the therapist's assessment, preferably based on the completed Mapping Form in P2, which the patient then agrees to/adjusts in the Treatment Plan.]

2. Send an SMS (at least once per week) to remind/support/encourage regarding one or more of the following. Agree with the patient on how and why SMS should be sent and document it in the Treatment Plan. Also, be clear about whether the patient is expected to respond via SMS or if dialogue is done in another way.
   a. General reminder to work on the treatment
   b. Log in and fill out forms, read and respond to homework reports
   c. Do a specific exercise
   d. Try a certain strategy in everyday life
   e. Record/note something relevant
   f. Allocate time to plan and prepare
   g. Allocate time to self-evaluate homework and treatment plan and decide on next steps.
   h. Advance notice that it's soon time to move on to the next part of the treatment
   i. Encourage the patient for accomplishments
   j. Other.

   [This is also important but generally, there should be fewer and less detailed SMS for light red patients.]

3. Telephone contact instead of, or in addition to, messages in the platform. Set up predetermined times (recorded in the treatment plan) and preferably several times per week and briefly describe what the phone calls will focus on (see suggestions below) and what will be addressed via phone versus in messages. Develop a contingency plan with the patient on how to proceed if the patient does not answer. Keep in mind that Problem Solving with the patient (see another point) can be a helpful support during phone contacts with patients.

IMPORTANT - if this means that the patient becomes less active in the treatment platform, consider that it affects the decision support tool's assessment in a negative direction. It is strongly recommended that the patient at least fills in the weekly ratings in the platform, or that the therapist does this together with or for the patient.

a) Go through something the patient finds difficult to understand or doesn't believe in or thinks they can handle.
b) Analysis of a specific problem/situation/behavior
c) Planning of a specific exercise/behavioral change
d) Real-time support while using a certain exercise/method
e) Follow-up on how a certain method, or the treatment as a whole, has progressed.
   (Can replace homework reports but should then be combined with the patient logging in and responding to forms before the call and then at least making some summary notes in the homework report at the end of the call that are submitted.)
f) Support to fill out forms, worksheets, or homework reports.
g) One or several "venting calls," meaning the patient gets to talk about how tough everything is
h) Patient provides module report over the phone instead of in the platform
i) Patient provides weekly report over the phone regarding e.g. status/techniques/interventions instead of regular module report
j) Go through homework or worksheets
k) Plan next week's work
l) Check the planning done in the platform/per message
m) Motivational phone call
n) Therapist summarizes module content over the phone (describe how, e.g. itemize what needs to be done, when, how long it's estimated to take)
o) Other

   [Shorter, more focused phone calls for light red. Maximum one phone call per week for light red.]

4. Allocate extra time to support, explain, and motivate in various ways, through messages, SMS, or phone calls depending on what suits best. This may involve:

a. Providing explanations and clarifications of module content/technical descriptions in messages. Sending links or additional materials to enhance understanding.
b. After active listening and reflection, addressing mistrust towards treatment techniques, principles, or models, or towards CBT/internet treatment in general.
c. Adjusting expectations and goals of the treatment. Investigate what expectations the patient has - both positive and negative - and goals, and assist them in adjusting them to align more with what can be achieved in treatment.
d. If the relationship between therapist and patient is assessed to be weak, spend more time on reflection, follow-up questions about things the patient perceives as important, and similar, with the primary purpose of improving the relationship.

[Maximum 15-20 minutes of extra time per week on this for light red.]

5. Adaptation, deepening, or broadening of techniques and treatment in general in one or more of the following ways. Note that Problem Solving together with the patient (see another point) can be a good basis for determining how a change should be made:
    a. Review what may cause a technique to not work well. This requires good knowledge of the CBT principles behind a particular technique and how it is described to the patient in the self-help material.
    b. Simplify a certain method by removing certain parts of it.
    c. Scale down the treatment to focus only on one or a few methods or modules. (If the patient finds it easier, it's okay to deactivate modules that are not needed.)
    d. Modify a certain method or use a related method.
    e. Provide the patient with new material. Clearly explain how the patient will receive the material and how homework reports are to be done. Also, describe how you as a therapist will follow up to ensure that the patient has received and started using the material.
    f. Provide advice and support for problems that currently occupy much of the patient's focus and energy, even if these do not have a clear connection to any method included in the treatment or to the patient's diagnosis. The purpose of this should not be to solve these problems in the long run but to temporarily bring about a change so that they hinder the treatment to a lesser extent and/or allow the patient to experience a positive change/relief in some context that increases motivation.
    Areas that may be addressed include:
        i. Relationship issues

        ii. Conflict at work or in other important contexts
        iii. Overload/time constraints
        iv. Specific avoidances or anxieties
        v. Other
    g. Other

    [Maximum 15-20 minutes of extra time per week on this for light red.]

6. Problem-solving via messages or phone calls, with or without the Problem Solving module, which includes a worksheet and a homework report that can be used. Always document the outcome of the problem-solving in the Treatment Plan worksheet. Problem-solving can involve the following areas:
    a. Finding more time for treatment
    b. Remembering to do various steps in the treatment
    c. How a specific exercise/technique can be managed despite being difficult/uncomfortable
    d. How a specific exercise/technique can be adapted
    e. Temporarily improving mood to be able to progress
    f. Increasing motivation for treatment and its goals in general
    g. Other

    [Same for light red.]

7. Transition entirely or partially to mail and paper to handle treatment materials. Examples are provided below.
    IMPORTANT - If this means that the patient becomes less active in the treatment platform, consider that it affects the decision support tool's assessment in a negative direction. It is strongly recommended that the patient at least fills in the weekly assessments in the platform, or that the therapist does this together with or for the patient.
    a. Mail the treatment plan, after agreeing with the patient on how it should look.
    b. Print out one or more modules or particularly important parts of texts and mail them to the patient.
    c. Allow the patient to fill out paper forms and/or homework reports.

    [Not suitable for light red, only after consultation with a supervisor.]
8. Change therapist. This is primarily done for practical reasons (e.g., if the current therapist has a serious lack of time) or because there are issues in the relationship between the patient and the therapist.

    [Only after consultation with a supervisor]

Temporarily involve a colleague or supervisor to go through something specific with the patient (often via phone but can also be through messaging). The main reason is that the substitute is an expert in a particular area or method, or matches the patient's profile/preferences in some important aspect.

[Only after consultation with a supervisor]

9. Other - determined during supervision!
