# Supplementary material for: Study protocol for a triple-blind randomised controlled trial evaluating a machine learning-based predictive clinical decision support tool for internet-delivered cognitive behaviour therapy (ICBT) for depression and anxiety
Source: Internet Interv. 2025 Mar 3;40:100816. doi: 10.1016/j.invent.2025.100816 (PMC11925161; doi:10.1016/j.invent.2025.100816)
Supplement: Supplement C — Interview Guide - Dark Red SOPHIA [file mmc3.pdf]

C. Interview Guide - Dark Red SOPHIA

Considerations Before and During the Interview:

- It's normal for a patient facing a major change to experience and show signs of some form of resistance. The art of working with resistance is to see it as a natural part of the change process and to explore the function of any resistance.
- Formulate obstacles and resistance in behavioral terms so that you and the patient get a concrete picture of what creates problems.
- When faced with resistance, alternate between being directive and change-focused and being listening, accepting, and validating. For example, it's often not very helpful to convey a lot of facts and emphasize the importance of doing homework. It can easily create more resistance. There may be nothing wrong with the intervention, but it can go wrong if it is communicated in a rigid and forced manner.
- The patient's thoughts about/attitudes toward treatment may be typical of the patient's issues (anxiety/depression). For example, feeling fear and apprehension about something new or feeling hopelessness and negativity.
- Explore contextual factors: what in the patient's situation may affect treatment? For example, the patient's partner may oppose the changes that treatment entails, substance abuse, etc.
- Are the problems due to deficiencies in the patient's skills?

The analysis you conduct during the phone interview lays the groundwork for what you should focus on in adaptation and extra support. The focus in behavioral analysis should be on the main diagnosis of the treatment program. What maintains treatment-disruptive behaviors and hinders functional behaviors? How can you help the patient move forward? Choose one or a couple of things at a time to focus on based on your analysis and the patient's preferences. It's probably not possible to address everything that is problematic all at once. A good basic principle can be to break it down into small steps with increasing difficulty.

It's important to note that the focus of treatment should be on the main problem - the patient sought help because they have issues with depression, panic disorder, or social phobia. Therefore, as a therapist, helping to keep the focus on that is validating. However, extra support means trying to address other problems so that they do not hinder treatment, and this can take quite a bit of time, such as how to implement treatment despite limited time and conflicts at home.

Many people have problems with activation and motivation, so there is great potential for these patients to benefit more from treatment with extra support. A patient categorized as light red or dark red by the decision support tool is therefore not discharged easily, even when one would normally assess that they might be too unmotivated or inactive. Instead, they are given "friendly leeches" to ensure they continue treatment. We can also offer much more support and more personalized treatment than those categorized as green by the decision support tool.

At the end of the interview or during a separate feedback session, when communicating actions/adaptations, it's helpful to take more responsibility than

usual and be more directive regarding what the patient should do regarding homework assignments, worksheets, etc., without spending too much time explaining the rationale. Provide clear instructions in the treatment plan or in a message. Some patients perceive rationale, text, and any required understanding as obstacles, so it's much better to initially focus on getting the patient to do rather than understand. Then, it's necessary to revisit understanding once the patient has started. Generally, it's good to break down what the patient needs to do into smaller parts and start with what is easiest/most feasible, then gradually increase the difficulty level.

Print out the following part of the document and make notes when speaking with the patient.

Participant ID:

**Interview Date (yyyy-mm-dd)**	
**Interviewer**	
**Participant's First Name**	
**Time at the Start of the Interview**	
**Time at the End of the Interview**	
**Total Duration**	

Before the interview:

First, take 5-10 minutes to review the patient's messages, worksheets, and assessments, and consider what you believe could be the problems or obstacles that classify the patient as dark red. Summarize your hypothesis here:

The phone interview should last between 20 - 45 minutes, and if you assess that more time is needed, it's better to schedule another call. Check with the patient how long you can talk to provide a clear framework. The interview is intended to build alliance and provide you with the opportunity to gather more information to make the analysis that forms the basis for what

you should focus on in providing extra support and adapting the treatment. The focus in the analysis and for the extra support should be on the diagnosis for which the patient is receiving treatment, so do not spend time analyzing and solving problems that are not related to the main diagnosis. Basic questions you want answers to in the interview are:

- What maintains non-functional behaviors, therapist-interfering behaviors, and avoidance?
- How can you help the patient plan and organize their work better?
- Does the patient need help breaking down the work into concrete sub-goals?
- Is it that the patient does their homework but in the wrong way?

Identify barriers, problem areas, and maintaining factors. You don't need to have a final plan or solution at the end of the call, but you can end the call by saying that you need to think through everything that has emerged and come back with a proposal on how to proceed. If there are many problematic issues, you need to choose one or a couple of things (at a time) to focus on based on the patient's wishes and your assessment.

Phone Interview:

Call at the agreed time:

“Hello, my name is [name], and I'm looking for NN. We've scheduled a time to talk now about your internet treatment. Are you undisturbed? I'll be asking about different parts of the treatment and how they're working for you.

Alternatively, you can say: We usually check in when you've made progress in the treatment/I've noticed that your ratings have been consistently high in the past few weeks...

I thought we'd talk for 20 - 45 minutes, does that work for you? (decide on an exact maximum time with the patient)"

Actively ask so that each heading below is covered. Ask about anything that is not obviously irrelevant.

If during the conversation you have gained a clear understanding of the problem and how it could be solved, you can suggest how you as the therapist can assist. Otherwise, say that you will compile all the information and ask to return with a proposal (see examples of actions on the last page).

I was thinking of starting by simply asking:

How do you feel about the technical/practical aspects of having internet-mediated treatment?

Ensure that the patient has good access to a computer with internet, logs in properly, and can get help if needed. Troubleshoot around this as needed.

DESCRIBE ISSUE

EXTRA SUPPORT/ACTIONS

For example, you could schedule a phone call with the patient to address any technical barriers that exist, or you could suggest where the patient can get technical support for issues that are beyond your expertise.

Agreed directly with the patient during the conversation.	Discussed with the patient and you should consider it and check with your supervisor.	Considered independently, not discussed with the patient, want to think more about it and/or discuss it in supervision first.

How is it possible to find time to work on the treatment?

How is it possible to plan and remember to do exercises/homework?

DESCRIBE THE ISSUE

(What is within the patient's control and what is beyond the patient's control in what they describe?)

PROPOSALS FOR EXTRA SUPPORT

It is crucial here to obtain a detailed description of how the patient currently manages their time, and proposals for solutions should be very specific. For example, you can assist the patient in creating a detailed weekly schedule outlining when and for how long they will work on various aspects of the treatment. You could suggest sending SMS reminders or calling the patient at one or more specific times to remind them to work on parts of the treatment or to check on their progress.

Agreed directly with the patient during the conversation.	Discussed with the patient and you should consider it and check with your supervisor.	Considered independently, not discussed with the patient, want to think more about it and/or discuss it in supervision first.

How do you feel about reading and understanding the module text?

How do you feel about writing module responses and messages?

DESCRIBE THE ISSUE

Identify any difficulties the patient may have with reading and writing.

SUGGESTIONS FOR ADDITIONAL SUPPORT

Provide suggestions on how the patient can divide the reading into small time intervals. Would it be easier for the patient to read the modules in PDF format? See if the patient can simplify the writing process in any way.

Agreed directly with the patient during the conversation.	Discussed with the patient and you should consider it and check with your supervisor.	Considered independently, not discussed with the patient, want to think more about it and/or discuss it in supervision first.

How is it going with the homework assignments (exposure/activation/cognitive processing/goal formulation, etc.)?

Identify both how much the patient practices and whether the patient is doing it correctly.

Also, connect it to the planning you have discussed earlier, as well as to the question below about whether the patient understands the purpose of a specific homework assignment.

DESCRIBE THE PROBLEM (If the patient seems to be working on their homework assignments, ask them to describe specifically what they are doing, so that you can determine if the patient might be doing something "wrong" when they expose themselves or activate themselves, etc.)

SUGGESTIONS FOR ADDITIONAL SUPPORT

Agreed directly with the patient during the conversation.	Discussed with the patient and you should consider it and check with your supervisor.	Considered independently, not discussed with the patient, want to think more about it and/or discuss it in supervision first.

Do you find the explanation of how the problem arises and persists clear? Do you think the explanations of why the methods in the treatment will make a difference are clear and seem to be accurate?

DESCRIBE THE PROBLEM

NOTE! Avoid getting into an argument with the patient. Listen primarily to the patient's perspective. Proposed comment if the patient has a different view of the basic model:
"Well, my perspective differs from yours, and this is something we could discuss further, which might make it easier for you to understand the treatment. Is that something you'd be open to?"

SUGGESTIONS FOR ADDITIONAL SUPPORT

Agreed directly with the patient during the conversation.	Discussed with the patient and you should consider it and check with your supervisor.	Considered independently, not discussed with the patient, want to think more about it and/or discuss it in supervision first.

How do you feel about the communication with me in the treatment?

A follow-up question could be about how the patient perceives your messages. Are they the right length, easy/difficult to understand, clear, etc.?

DESCRIBE THE PROBLEM

Transition to discussing possible additional support: As you explain this, I'm thinking maybe there's a different approach we could try.

SUGGESTIONS FOR ADDITIONAL SUPPORT

Agreed directly with the patient during the conversation.	Discussed with the patient and you should consider it and check with your supervisor.	Considered independently, not discussed with the patient, want to think more about it and/or discuss it in supervision first.

What other factors about you as a person or in your life affect how you work with the treatment?

DESCRIBE THE PROBLEM

If the patient has difficulty responding, you can assist by saying, for example:

"You mentioned in one of your messages that / I got the impression from one of your messages that you weren't so keen on / You really seemed to dislike the idea of..."

SUGGESTIONS FOR ADDITIONAL SUPPORT

Agreed directly with the patient during the conversation.	Discussed with the patient and you should consider it and check with your supervisor.	Considered independently, not discussed with the patient, want to think more about it and/or discuss it in supervision first.

What currently affects how you feel, either related to your personality or aspects of your life?

For example, internal issues like other anxiety or worries that are not the focus of treatment, or personality traits like perfectionism, neurodevelopmental disorder diagnoses, etc. External issues like relationship problems, conflicts at work or with family/friends, unemployment, issues with relatives/children, financial stress, etc.

DESCRIBE THE PROBLEM

SUGGESTIONS FOR ADDITIONAL SUPPORT

Agreed directly with the patient during the conversation.	Discussed with the patient and you should consider it and check with your supervisor.	Considered independently, not discussed with the patient, want to think more about it and/or discuss it in supervision first.

How do you perceive your motivation to engage in treatment?

Examples of factors that can affect the patient's motivation include feeling much better, not believing that it will help, finding it too difficult, or being disrupted by other events in the patient's life.

DESCRIBE THE PROBLEM

SUGGESTIONS FOR ADDITIONAL SUPPORT

Agreed directly with the patient during the conversation.	Discussed with the patient and you should consider it and check with your supervisor.	Considered independently, not discussed with the patient, want to think more about it and/or discuss it in supervision first.

How does any medication affect the treatment? (skip if not applicable)

Follow-up questions: Is there any general concern about medication? Any ongoing changes in the patient's medication? What is the patient's current medical contact?

Do you have any other medical care or interventions that currently affect you and this treatment?

DESCRIBE THE PROBLEM

SUGGESTIONS FOR ADDITIONAL SUPPORT

Agreed directly with the patient during the conversation.	Discussed with the patient and you should consider it and check with your supervisor.	Considered independently, not discussed with the patient, want to think more about it and/or discuss it in supervision first.

What else might be affecting your internet treatment that we haven't discussed?

DESCRIBE THE PROBLEM

SUGGESTIONS FOR ADDITIONAL SUPPORT

Agreed directly with the patient during the conversation.	Discussed with the patient and you should consider it and check with your supervisor.	Considered independently, not discussed with the patient, want to think more about it and/or discuss it in supervision first.

Suggestion for when you need to get back to the patient: "I would need some time to think about how I can best assist you based on the conditions we have in this treatment. But can't we schedule a new time to talk in a couple of days, so I can think about this calmly, and you can too, and then we can see how to proceed?"

Note below things to find out, thoughts you have received, etc., as well as the new time when you will be in touch.

Seek supervision and go through your questions and considerations.

Summary

Write down the results of the interview/behavioral analysis. Establish a clear treatment plan. It should always be added to the worksheet in the extra module Treatment Plan, and the patient should be informed via a message that the plan is available in the worksheet in the Treatment Plan module. If the patient has difficulty navigating the platform, an alternative may be to also send the text of the treatment

plan to the patient in a message. The plan should preferably be reviewed at each contact and revised continuously. All changes are entered into the worksheet.

The plan should be very instructional, specifying exactly when and for how long the patient should work on homework/exposure/etc., when and how to contact the therapist, and which exercises the patient should work on. Even if the plan only includes, for example, a change in the order of modules or weekly phone calls, this should be added to the worksheet in the Treatment Plan module.

The interview notes (paper) are considered working material and can be discarded once the treatment plan is established.

The worksheet serves three purposes:

- To communicate with the patient about the agreed-upon plan.
- To communicate with other therapists who may step in for you.
- To document the extra support provided so that it can be systematically analyzed as part of the study.

Refer to the list "**Examples of Actions for Customizing Treatment for Light and Dark Red Patients**" in the SOPHIA Quick Guide Decision Support for further guidance.
