# Supplementary material for: Study protocol for a triple-blind randomised controlled trial evaluating a machine learning-based predictive clinical decision support tool for internet-delivered cognitive behaviour therapy (ICBT) for depression and anxiety
Source: Internet Interv. 2025 Mar 3;40:100816. doi: 10.1016/j.invent.2025.100816 (PMC11925161; doi:10.1016/j.invent.2025.100816)
Supplement: Supplement D — Therapist and patient questionnaires [file mmc4.pdf]

D. Therapist and patient questionnaires

Contents

1. The Internet Psychiatry Clinic’s standard evaluation questionnaire (including CSQ-8)
2. Patient perspective Questionnaire
3. Treatment adherence, belief, and knowledge
4. Therapist Questionnaire - (TAU)
5. Therapist Questionnaire – Decision Support Tool (DST)

1. The Internet Psychiatry Clinic’s standard evaluation questionnaire (including CSQ-8)

Version 3
1. How would you rate the quality of your treatment?
Excellent
Good
Decent
Poor
2. Did you receive the type of treatment you had hoped for?
No, absolutely not
No, not really
Yes, for the most part
Yes, absolutely
3. To what extent did the treatment meet your needs?
It met nearly all of my needs
It met most of my needs
It met only a few of my needs
It did not meet my needs at all
4. If a close friend of yours needed similar help, would you recommend the same type of treatment you received?
No, absolutely not
No, I don't think so
Yes, I think so
Yes, absolutely

5. How satisfied are you with the extent of the help you received from us?
Quite dissatisfied
Indifferent or slightly dissatisfied
Mostly satisfied
Very satisfied
6. Has the treatment helped you adopt a better approach to your problems?
Yes, it has helped me a great deal
Yes, it has helped me somewhat

No, it hasn't really helped me
No, it seems to have worsened the situation
7. Overall, how satisfied are you with your treatment?
Very satisfied
Mostly satisfied
Indifferent or slightly dissatisfied
Quite dissatisfied
8. If you needed help in the future, would you seek our services again?
No, absolutely not
No, I don't think so
Yes, I think so
Yes, absolutely
9. How easy was it to access and start the treatment?
Very difficult
Difficult
Neither easy nor difficult
Easy
Very easy
10. Have you been treated kindly and respectfully?
No, absolutely not
No
Yes, but there were some shortcomings
Yes
Yes, absolutely
11. How much of all the text in the treatment did you read in total?
Less than 25%
25%
50%
75%
More than 75%
12. How actively did you work on the various homework assignments and try out what was covered in the treatment? Exclude reading assignments and estimate how much you did of other exercises.
Less than 25%
25%
50%
75%
More than 75%
13a. Did you terminate the treatment prematurely (before the agreed treatment time, regardless of how many modules/steps were completed)?
No
Yes
13b. If yes, select one or more options that apply to you.
Because I felt better and didn't need more help
Because I didn't have time for the treatment
Because I didn't feel the treatment suited me
Because the treatment was too difficult and challenging
Because I felt worse and needed different help

Due to other reasons
14. How much of the text in each module did you read before starting the homework assignments?
Nothing or almost nothing
Significantly less than half
About half
Significantly more than half
All that was available to read
14a. Have you encountered any significant problems with the treatment?
No
Yes
14b. If yes, select one or more options that apply to you.
I had too little time and couldn't keep up
I felt that the treatment wasn't helping me
I didn't think the treatment addressed the problems I needed help with
I didn't have the energy to complete the treatment
I was dissatisfied with the treatment format
I found it difficult to understand how to proceed
I felt I received too little support from the therapist
I found the technical aspects of the treatment platform cumbersome
15a. Has this treatment led to any negative experiences or events?
No
Yes
15b. If yes, please describe in what way:
16. Have you started or undergone any other psychological treatment during the internet treatment?
Select one or more options that apply to you:
No, I have not had any other psychological treatment
Yes, I have been in psychodynamic psychotherapy
Yes, I have been in CBT treatment
Yes, I have been in another type of psychological treatment.
Please describe:
17. Have you taken medication for the condition you were working on during the internet treatment?
Select the option that best applies to you:
No, I have not taken any medication for these issues
Yes, I have been on the same medication and dosage as before
Yes, I have been on the same medication but changed the dosage
Yes, I started medication during this time
Yes, I have changed medications
Yes, but I have stopped medication
18. Did you feel that anything was missing in the treatment or therapist contact? Was there anything in the treatment that you found less beneficial?

19. What in the treatment did you benefit from the most? Write what you found most beneficial at the top.
19a. I benefited most from:

19b. I benefited next most from:
19c. I then benefited from:

2. Patient perspective Questionnaire

How have you experienced the treatment?
1. I feel that what I did in the treatment has given me new ways to look at my problems.
Never Rarely Occasionally Quite often Often Very often Always
2. I feel that what was included in the internet treatment was important for me to work on.
Never Rarely Occasionally Quite often Often Very often Always
3. I feel that my therapist and I agreed on what was important for me to work on in the treatment.
Never Rarely Occasionally Quite often Often Very often Always
4. I feel that my therapist and I worked on my problems in an appropriate way during the treatment.
Never Rarely Occasionally Quite often Often Very often Always
5. I feel that the structure of the internet treatment was well adapted to my conditions and needs.
Never Rarely Occasionally Quite often Often Very often Always
6. I feel that the communication with the therapist was well adapted to my conditions and needs.
Never Rarely Occasionally Quite often Often Very often Always
7. Would you have managed the treatment as well without the therapist's support?
I would have managed the treatment better without therapist support
I would have managed the treatment just as well without therapist support
I would have managed the treatment slightly worse without therapist support
I would have managed the treatment worse without therapist support
I would have managed the treatment much worse without therapist support
8. How much overall help did you feel you received from the contact with the therapist?
No help at all
Not much help
Quite a lot of help
A lot of help
Very much help
9. When you had problems or questions, how often did you ask the therapist about them?
Never
Only a few times
About half the time
Almost always
Every time
(Didn't have any problems or questions)
10. Are you satisfied with how quickly the therapist responded?
Very dissatisfied
Dissatisfied
Neither satisfied nor dissatisfied
Satisfied
Very satisfied

11. Was it easy or difficult to understand what the therapist wrote?
Very difficult
Difficult
Neither particularly easy nor difficult
Easy
Very easy
12. What did you feel was missing in your contact with the therapist?
13. Do you think the communication with the therapist has been superficial or personal?
Very superficial
Superficial
Neither particularly superficial nor personal
Personal
Very personal
Continued Care Needs
1. Do you feel that you have received sufficient help from the treatment you underwent regarding the problem you sought help for?
Fully sufficient
Almost sufficient
Far from sufficient
Not at all sufficient
2. Do you still need help with the problem you sought assistance for?
Yes
Unsure, but I believe it's needed
Unsure, but I believe it's not needed
No
3. Will you now or soon seek additional care for the problem you sought help with from us?
No (and I currently have no other care for this problem)
No (but I will continue with the care/treatment I already have without changing it)
Yes, I will seek more care or change the care I already have (Also answer question 4)
4. What type of care will you seek?
Medical treatment (other than what I already have)
Medical treatment (same as I have now but with changes e.g., in dosage)
Cognitive behavioral therapy (CBT)
Other psychotherapy than CBT (specify type of therapy)……………………………
Internet treatment
Other care (describe): …………………………………………………
Not sure right now, but will contact healthcare to consult
5. Will you now or soon seek care for any other problem?
No
Yes (Describe for which problem(s) and what type of care): ___________________

3. Treatment adherence, belief, and knowledge

How has the treatment been going recently?

1. Choose the option that best describes how you've worked with the treatment in the past two weeks. This could include reading treatment text, doing exercises/homework in your daily life, or consciously trying new approaches as suggested by the treatment.

□ Not at all or very little
□ I have tried but am unsure if I understood how to do it
□ I have partly done it differently than suggested by the treatment, or used methods other than those in the treatment
□ I have both continued with things I started in previous treatment weeks and tried new things in the treatment
□ Only with things from previous treatment weeks
□ Only with new things

2. Have you been able to work on the treatment as much as you wanted/planned in the past two weeks?

□ Yes, with a good margin
□ Yes, about as much as I wanted/planned
□ Yes, but barely
□ No, lack of time or unexpected events have prevented me
□ No, I have not wanted to work on the treatment or have felt significant hesitation towards it

How has the following changed for you during the past week in treatment, compared to before treatment and during previous treatment weeks?

3. After the past two weeks of treatment, has your belief that the treatment is suitable for you and your situation changed?

□ Believe in it much more than before
□ Believe in it more than before
□ Believe in it slightly more than before
□ Unchanged
□ Believe in it less than before

4. How much has what you've read and done in the past treatment week changed your understanding of depression/social phobia/panic disorder, how you view your problems with depression/social phobia/panic disorder, and how they can be managed?

□ Unchanged
□ Changed slightly
□ Changed somewhat
□ Changed significantly
□ Changed very significantly

4. Therapist Questionnaire (TAU)

Here are 17 questions about the questionnaire, clinical procedures, and supervision.

1. What is your general opinion of the questionnaire?

o I am very negative towards it
o
o
o Neutral
o
o
o I am very positive towards it

2. How often do you trust the information in the questionnaire?

o I never trust the information in the questionnaire
o
o
o I trust it about half of the time
o
o
o I always trust the information in the questionnaire

3. When assessing a patient's condition and writing a response, how useful do you find the information in the questionnaire?

o Not useful at all
o
o
o Moderately useful
o
o
o Very useful

4.How comprehensible do you find the information in the questionnaire?

o Impossible to understand
o
o
o Moderately easy to understand
o
o
o Very easy to understand

5. Do you think the information in the questionnaire is adequately detailed?

o Far too little detail
o
o
o Just the right level of detail
o
o
o Far too much detail

6. Do you believe that using the questionnaire improves or worsens the treatments you conduct?

o Treatment outcomes become much worse

o
o
o Makes no difference
o
o
o Treatment outcomes become much better

7. How do you perceive the guidance from the questionnaire on what you as a therapist should do during treatment?
o Too weak
o
o
o Moderate
o
o
o Too strong
8. What problems have you experienced with the questionnaire or the procedures related to it? (Please provide a brief description.)
9. How can the questionnaire or the procedures surrounding it be improved? What suggestions do you have for improvement? (Please provide a brief description.)
10. What would you like to remove or add to the questionnaire? (Please provide a brief description.)
11. How much do you feel these factors influence treatment? (Allocate 100% among the following options)
Questionnaire
Supervision
Treatment materials (i.e., text, worksheets, etc. in the modules)
Personal clinical judgment
Decision support
12. How much do you feel you have learned about CBT in general?
o Not at all
o
o
o Quite a lot
o
o
o Very much
13. How much do you feel you have learned about iCBT specifically?
o Not at all
o
o
o Quite a lot
o
o
o Very much
14. Do you recommend using the questionnaire?
Yes/No

If yes, provide up to three examples of when: (free text)
15. What is your general opinion of the supervision?
o I am very negative towards it
o
o
o Neutral
o
o
o I am very positive towards it
16. What is your general opinion of the clinical procedures?
o I am very negative towards them
o
o
o Neutral
o
o
o I am very positive towards them
17. How can the supervision and/or the clinical procedures be improved? What suggestions do you have for improvement? (Please provide a brief description.)

5. Therapist Questionnaire – Decision Support Tool (DST)

Here are seventeen questions about the decision support tool, clinical routines, and the guideline.
1. What is your general opinion of the decision support tool?
o I am very negative about it
o
o
o Neutral
o
o
o I am very positive about it
2. How often do you rely on the information/predictions from the decision support tool?
o I never rely on the information/predictions from the decision support tool
o
o
o I rely on it in about half of the cases
o
o
o I always rely on the information/predictions from the decision support tool
3. When assessing a patient's situation and writing a response - How useful do you find the information/predictions in the decision support tool?
o Not useful at all
o
o
o Moderately useful
o

o
o Very useful
4. How understandable do you find the information/predictions in the decision support tool?
o Impossible to understand
o
o
o Quite easy to understand
o
o Very easy to understand
5. Do you think the information/predictions in the decision support tool are adequately detailed?
o Way too little detail
o
o
o Just the right level of detail
o
o
o Way too much detail
6. Do you believe that using the decision support tool improves or worsens the treatments you conduct?
o Treatment outcomes are much worse
o
o
o Makes no difference
o
o
o Treatment outcomes are much better

7. How do you perceive the guidance from the decision support tool on what you as a therapist should do in the treatment?
o Too weak
o
o
o Just right
o
o
o Too strong
8. What problems have you experienced with the decision support tool or the routines around it?
(free text) (briefly describe)
9. How can the decision support tool or the routines around it be improved? What suggestions do you have for improvement?
(free text) (briefly describe)
10. What would you like to remove or add to the decision support tool?
(free text) (briefly describe)
11. How much do you think these factors influence the treatment?
(distribute 100% among the following options)

Questionnaire
Supervision
Treatment materials (i.e., text, worksheets, etc. in the modules)
Personal clinical judgment
Decision support
12. How much do you feel you have learned about CBT in general?
(free text) (write briefly)
13. How much do you feel you have learned about iCBT specifically?
(free text) (write briefly)
14. Have you, based on your clinical assessment, deviated from what the decision support recommended?
Yes/No
If yes, provide up to three examples of when you clearly did something different than what the decision support recommended: (free text)
15. What is your general opinion about the cheat sheet and the clinical routines?
o I am very negative about it
o
o
o Neutral
o
o
o I am very positive about it
16. What is your general opinion about the guidance?
o I am very negative about it
o
o
o Neutral
o
o
o I am very positive about it
17. How can the guidance and/or the clinical routines be improved? What improvement suggestions do you have?
(free text) (write briefly)
