# Supplementary material for: Study protocol for a triple-blind randomised controlled trial evaluating a machine learning-based predictive clinical decision support tool for internet-delivered cognitive behaviour therapy (ICBT) for depression and anxiety
Source: Internet Interv. 2025 Mar 3;40:100816. doi: 10.1016/j.invent.2025.100816 (PMC11925161; doi:10.1016/j.invent.2025.100816)
Supplement: Supplement E — SERA study protocol [file mmc5.pdf]

# Ansökan om etikprövning – *Bilaga 3*

# Forskningsplan avsedd för fackmän

## Background

Depression, social anxiety disorder, and panic disorder are prevalent and lead to individual suffering and negative societal consequences (1). Cognitive behavioral therapy (CBT) has strong empirical support but still, 30-60% of patients end up as non-responders. Personalized medicine aims to identify patients that are suitable for a certain treatment or need a certain adaption within a treatment. All types of data can be used for the purpose of identifying patient needs and guide clinical decisions (2).

A 'Learning Machine' is a combination of a number of machine learning/artificial intelligence (AI) algorithms. It can use a multitude of data sources and learn from a large set of example cases, for example patients receiving a treatment, and then applying what it has learnt to predict outcome for a new patient. Machine learning/AI has recently been introduced in psychiatric research, for example in suicidality prediction and using fMRI to distinguishing CBT responders from non-responders (3), in analysis of free speech to predict later development of psychosis (4), to estimate long-term severity of depression (5), and to identify patients likely to respond to a specific antidepressant (6).

A vast majority of traditional prediction research focuses on finding factors that predict for example treatment outcome. The strength of a Learning Machine is that it combines many predictors, also rather weak ones, and makes predictions on the individual level. However, to actually become useful in regular care, two criteria must be fulfilled: (1) the prediction for a patient needs to be strong enough to base important clinical decisions on and (2) the prediction must be presented to the clinician in a way that is easy to grasp, acceptable and suitable within the clinical context. So far, not even studies using AI/machine learning to classify psychiatric patients have actually taken the next step and developed a decision support tool (DST), i.e. a system presenting predictions for therapists and give them guidelines on how to interpret and act on these predictions, and then tested its ability to increase quality of care.

Internet-based Cognitive Behavioral Therapy (ICBT) with brief therapist support emerged in the late 90-ies and has rapidly received empirical support for a wide range of conditions (7). The primary investigator in this project has since 1999 been a pioneer in in evaluating and implementing ICBT in regular care and Sweden is one of the leading countries in this field, where the Internet Psychiatry Clinic (www.internetpsykiatri.se) in Stockholm is a very successful implementation. ICBT is a highly suitable context for developing and testing a DST powered by a Learning Machine. It collects a lot of data, is highly standardized and thus more predictable, and is easy to integrate with a digital treatment platform.

The idea of achieving strong enough predictions to base clinical decisions on might seem like a utopia, even with the computational power of a Learning Machine. If the goal is to use baseline data to match a patient to a treatment, previous failures to show coherent results

are indeed discouraging (for example 8, 9). However, the picture becomes brighter when data from the initial parts of treatment is added. Continuous monitoring of symptoms has been used to detect patients at risk of being a non-responder in a more reliable way than psychotherapist's own predictions (10), and has led to fewer failed treatments (11). Using early weekly symptom ratings in ICBT has also been shown to be successful in predicting treatment failures (12).

A predictive DST combined with a treatment that has the capability of quickly adapting to the clinical needs of an identified, probable non-responder thus has the potential to avoid treatment failures and increase overall response rates. We call this method an Adaptive Treatment Strategy. Besides the above mentioned advantages in integrating ICBT and a decision support tool, ICBT provides good opportunities to increase the "treatment intensity" during treatment since it starts from a low intensity (10-15 minutes of therapist time each week) which can be increased by for example intensifying therapist support and/or provide it via telephone or add new treatment modules.

Personalized medicine is the science of individualized care and aims to identify which patient would benefit from which type of care. Internet-based Cognitive Behavioral Therapy (ICBT) in combination with Artificial Intelligence (AI) and machine learning provides an opportunity to start implementing personalized medicine in psychiatric care. Previous studies using AI/machine learning within the context of personalized medicine have only evaluated the predictive capacity, without actually testing if the categorization of patients can be transformed into a tool that results in better treatments.

Continuous monitoring of symptoms to some extent predicts outcome in psychological treatment. We have previously used an Adaptive Treatment Strategy in a randomized clinical trial to identify patients in ICBT for insomnia at risk of being non-responders and considerably increased positive outcome for those by providing enhanced ICBT (13). However, the semi-manual classification routine consumed valuable therapist time and its predictive power leaves room for improvement. A so called Learning Machine combines a range of AI/machine learning algorithms to use a wide range of data sources to learn from a large set of examples (patients) and applies this knowledge on a new patient, in order to for example predict final outcome (14). Learning Machines can often outperform traditional prognostic methods but so far their actual usefulness in a clinical setting have not been tested in a RCT.

In an ongoing collaboration between the Internet Psychiatry Clinic, KI, and KTH we have used 5500 historical patients having received ICBT for depression, social anxiety or panic disorder to train a Learning Machine to use data from baseline and early on in treatment (primarily week 1-4) to predict a patient's end state. The primary outcome we have predicted is patients being non-responders or deterioraters, since this is the most important group to intervene on if using an Adaptive Treatment Strategy. This has then been used as a base for a DST.

During the spring of 2020 we have developed a first version of this DST giving ICBT therapist at the Internet Psychiatry Clinic feedback on their patients' predicted end state. We involved the users (therapists) in the development process and in a pilot trial with actual patients and evaluated its acceptability and perceived usefulness before finalizing its design. This pilot

study needs to be followed up by a randomized controlled trial to evaluate the clinical effects of this tool.

## Aim & Research questions

The overall aim is to test the actual clinical benefits of an AI-based DST being used as the core component in a so called 'Adaptive Treatment Strategy' and to evaluate how the DST affects therapists and their patients with depression, social anxiety, or panic disorder during 12 weeks of ICBT. The effect of this is expected to be fewer treatment failures, larger overall reductions of symptoms, more efficient use of therapists' time, and less need for further treatment.

More specifically, the project aims to evaluate ICBT with a DST for depression, panic disorder and social anxiety (the DST arm) compared to ICBT using only a traditional, detailed therapist manual and making, but not showing, predictions (the TRAD arm) concerning the following areas:

1. The DST will result in larger symptom reductions (Primary Hypothesis) and fewer actually failed treatments, (i.e. being neither a responder nor a remitter), among patients predicted to fail comparted to TRAD.
2. The overall reduction of the patient's disorder specific symptoms and number of failures will be larger for DST than for TRAD.
3. The DST will make therapists more time efficient, defined as the ratio of 'decrease in symptoms / therapist time spent on patient' compared to TRAD.
4. The DST will have superior outcomes compared to TRAD on the following:
    a. Level of everyday functioning
    b. Health related quality of life
    c. Treatment adherence/compliance
    d. Patient satisfaction
    e. Number of Adverse Events experienced by the patient
    f. Need for further treatment
    g. Sudden symptom changes
5. For therapists, the DST will, compared to TRAD and the therapist manual used there, result in:
    a. A more positive experience of using the DST / the therapist manual, including higher perceived helpfulness and credibility and the overall experience of supervision, clinical routines, and guidance of their clinical decisions
    b. More and longer contacts (messages, phone/video calls) with patients, more treatment adaptions made, and more detailed advice and feedback for those patients predicted to fail.
    c. The same level of contacts, adaptions and details for patients predicted to succeed.

## Method

### Design

This study is designed as a triple blind randomized controlled trial, where half of the therapists allocated to DST and the other half to TRAD. The therapists and patients will be blind to what group they are allocated to and the post-treatment assessors will also be blind to what group the patients belong. The therapists and patients will know that they are

randomized, but will be informed that the study concerns two different types of treatment structure and supervision, and will thus be blind to the true purpose of randomization. After randomization both groups will be informed that they are in the experimental group to further ensure that they are blind, therapists in TRAD will be informed that the other arm receives a much simpler therapist manual and half the amount of supervision. The trial will follow the guidelines of Good Clinical Practice adapted for psychological treatment.

### Procedure

We will start with recruitment of novice therapists (see below for details). Followed by a general training for all therapists in the basics of the treatment programs and routines. Here therapists learn to use the general therapist manual. After the general training a suitability testing of all therapists is performed to ensure that they have an adequate level of expertise in ICBT. After training therapists are randomized, 1:1 ratio, to either ICBT with DST or ICBT TRAD. The randomization is handled by an independent party (Karolinska Trial Alliance (KTA)) and will be on the therapist level. Separate, specific training of the two experimental groups, where both groups will be informed that they are in the “active” group. One group will be trained in how to use the DST and the other group will receive further training in the clinical routines and treatment structure (they will again be informed that this specific treatment structure and manual is what is being evaluated). Both groups receive a specific therapist manual adapted for DST or TRAD.

Recruitment and assessment of patients is done parallel to therapist recruitment and training. Randomization of patients is done to the two treatment arms, 1:1 ratio. After randomization patients receive 12 weeks of guided ICBT with a DST or guided ICBT TRAD. Patients answer online questionnaires at screening, pre-treatment, weekly measures during treatment, post-treatment and at follow-up assessment.

### Participants and recruitment

Both therapists and patients are participants in this trial. The trial will consist of 10-40 novice therapists, primarily students in the final year of the 5-year clinical psychologist program, with basic education in CBT but little or no experience of ICBT. Each will treat 5-25 patients (total patient n=250 - 700).

The therapists will be recruited nationally in Sweden from different clinical psychologist programs thru advertising and contacts with key persons at these universities. If necessary additional therapists will be recruited among students who are finishing the two-year basic training program in CBT, plus among intern psychologists (‘PTP-psykolog’) at the psychiatric clinic at Psykiatri Sydväst in Stockholm. The patients will be recruited through the Internet Psychiatry Clinic routine intake plus through advertising on the web, for example on www.studie.nu, and in traditional media.

A large number of ICBT studies have successfully used therapists with this level of competence. None of the therapists (or patients) will be aware of the randomization. Therapists are informed they will have the opportunity to learn ICBT within the context of a clinical trial evaluating slightly different versions of ICBT, and receive a diploma and in some cases a small monetary reimbursement or the possibility to take the course and treat the ICBT patients during office hours.

The patients in this setting will also be recruited nationally and assessed via internet and structured, diagnostic video- or telephone interviews according to well established procedures from previous ICBT-trials. Its content will mimic the assessment at the Internet Psychiatry Clinic.

*Inclusion criteria:*

- 18 years or older.
- Social anxiety, panic disorder or depression diagnosis.
- Stable or no antidepressant medication for at least 2 months.
- No diseases, disorders, or substance abuse that required other, immediate attention (e.g., severe depression or suicidality).
- Available time for treatment and acceptance of its format.
- Proficient in Swedish.
- No ongoing CBT.

*Exclusion criteria:*

- Not available for assessment and diagnostic telephone interview.
- Will not be in Sweden during the treatment period.
- Not able to receive text messages on a Swedish mobile phone.
- Not proficient in Swedish.
- No access to computer and internet.
- Not able to set aside about one hour a day to work on treatment.
- Bipolar disorder, if seeking depression treatment

All included patients will receive an ICBT-program for depression, social anxiety, or panic disorder. Patients log in to the secure, web based, online treatment platform to start the 12-week treatment program consisting of 10 modules. Every week patients receive a new module, read the text in the module, work on their own with exercises and work sheets. When a module is finished patients receive feedback and encouragement from their therapist through text messages. They can also ask questions to the therapist via text messages. When a module is finished the therapist assigns a new module. Therapists also monitor the patients' weekly symptom ratings to assess suicidality and large increases of symptoms.

All therapists receive a specific therapist manual, including recommendations of how much time to spend on each patient. Clinical psychologist with experience of ICBT will provide supervision via comments within the treatment platform and in weekly group format supervision via secure video conferencing, including only therapists from the same study arm. Therapists can also consult the supervisor by phone if necessary. Therapists continuously follow the therapist manual that they have been trained in during the introductory course. In the therapist manual all clinical routines and scenarios are described.

*Decision Support Tool (DST) group*

Therapists randomized to use the DST will have instructions on when and how to act on the feedback from the DST, for example by increasing intensity or type of support. The DST group will, mostly, during the first half of the treatment period receive guidance from the DST regarding the patient's expected final outcome which is defined as successful (the patient is either in remission, defined as below the previously established limit for diagnosis-

specific symptom scale and/or is a responder, i.e. has reduced symptoms by at least 50%) or failed (others). The primary indicators in the DST are colors that show that the patient's treatment is likely to be successful (green), likely to fail (red), very likely to fail (dark red), or that the prediction is too uncertain to say anything about the final outcome (yellow). In the case of red and green indicators, the therapist makes adjustments to the treatment based on clinical assessment, more detailed indicators in the DST and the specific manual that describes how the DST is to be interpreted. For red patients, it could for example be making a telephone assessment of the patient's problems and difficulties with the treatment, adapting parts of the content and how the treatment is given and providing more and/or other type of treatment support. Dark red means that the therapist, regardless of his or her own clinical assessment of the patient's situation, must make a telephone assessment and some form of adjustment. For green patients, the therapist is recommended to answer more concisely to messages from the patient, support the patient's independent work and monitor that the patient's progress remains good. The therapist's clinical assessment of what the patient needs may take precedence over these recommendations from the DST and the manual.

*Control group*

As described above the control group will also be included in teams with only therapists in the same experimental condition and receive a specific therapist manual with detailed instructions on how to guide patients through the treatment program and how to handle different clinical scenarios. Therapists will, through the manual and in supervision, be encouraged to assess how the patients are doing and to monitor their progress.  As noted above they will be informed that they are in the active experimental group and that this study is evaluating the effect of their specific therapist manual and type of supervision. This procedure is expected to lower the risk that these therapists suspect they are in the control group.

### Statistical Analyses

Primary analyses will be performed according to the intent-to-treat principle with Longitudinal Multilevel Modelling (‘Mixed Models’) or GEE including baseline covariates to control for non-random missingness. The power analysis is based on the primary hypothesis concerning differences in symptom reduction between DST and TRAD for patients being predicted to fail. Our previous proof-of-concept trial (13) shows that patients that during week four are predicted to fail and then receives enhanced ICBT increase their outcome (Cohen’s d=0.53) compared to those predicted to fail that stays on standard ICBT. To be conservative, we now estimate the effect the decision support tool to be d=0.40, which with a power of 80% and an data attrition of 20% would require 250 patients. Mainly because some secondary hypotheses are assumed to need more power and partly because the estimated effect is somewhat unsure and the design with randomization on the therapist level might can decrease power further (15), we will perform an interim, renewed power analysis after 250 patients have finished treatment. Depending on this analysis we can, if needed, include up to a total of 700 patients.

## References

1. Socialstyrelsen (2013). Nationell utvärdering 2013 – vård och insatser vid depression, ångest och schizofreni Rekommendationer, bedömningar & sammanfattning. Stockholm.

2. Khoury MJ, Ioannidis JP. Big data meets public health: Human well-being could benefit from large-scale data if large-scale noise is minimized. Science (New York, NY). 2014;346(6213):1054.

3. Passos IC, Mwangi B, Kapczinski Fv. Big data analytics and machine learning: 2015 and beyond. The Lancet Psychiatry. 2015;3(1):13-5.

4. Bedi G, Carrillo F, Cecchi GA, Slezak DFn, Sigman M, Mota NlB, et al. Automated analysis of free speech predicts psychosis onset in high-risk youths. Npj Schiz. 2015;1.

5. Kessler R, van Loo H, Wardenaar K, Bossarte R, Brenner L, Cai T, et al. Testing a machine-learning algorithm to predict the persistence and severity of major depressive disorder from baseline self-reports. Molecular psychiatry. 2016.

6. Chekroud AM, Zotti RJ, Shehzad Z, Gueorguieva R, Johnson MK, Trivedi MH, et al. Cross-trial prediction of treatment outcome in depression: a machine learning approach. The Lancet Psychiatry. 2016.

7. Andrews G, Cuijpers P, Craske MG, McEvoy P, Titov N. Computer Therapy for the Anxiety and Depressive Disorders Is Effective, Acceptable and Practical Health Care: A Meta-Analysis. PLoS One. 2010;5(10):e13196.

8. Knopp J, Knowles S, Bee P, Lovell K, Bower P. A systematic review of predictors and moderators of response to psychological therapies in OCD: Do we have enough empirical evidence to target treatment? Clin Psychol Rev. 2013;33(8):1067-81.

9. Schneider RL, Arch JJ, Wolitzky-Taylor KB. The state of personalized treatment for anxiety disorders: A systematic review of treatment moderators. Clin Psychol Rev. 2015; 38:39-54.

10. Lambert MJ. Progress feedback and the OQ-system: The past and the future. Psychotherapy. 2015;52(4):381.

11. Shimokawa K, Lambert MJ, Smart DW. Enhancing treatment outcome of patients at risk of treatment failure: meta-analytic and mega-analytic review of a psychotherapy quality assurance system. Journal of consulting and clinical psychology. 2010;78(3):298.

12. Forsell E, Isacsson N, Blom K, Jernelöv S, Ben Abdesslem F, Lindefors N, Boman M, & Kaldo, V. Predicting treatment failure in regular care Internet-Delivered Cognitive Behavior Therapy for depression and anxiety using only weekly symptom measures. Journal of Consulting and Clinical Psychology. 2019; 88(4), 311–321.

13. Forsell E, Jernelöv S, Blom K, Kraepelien M, Svanborg C, Andersson G, et al. Proof of Concept for an Adaptive Treatment Strategy to Prevent Failures in Internet-Delivered CBT: A Single-Blind Randomized Clinical Trial with Insomnia Patients. American Journal of Psychiatry. 2019 Apr 1;176(4):315–23.

14. Boman M, Ben Abdesslem F, Forsell E, Gillblad D, Görnerup O, Isacsson N, et al. Learning machines in Internet-delivered psychological treatment. Progress in artificial intelligence. 2019;8(4):475–85.

15. Magnusson K, Andersson G, Carlbring P. The consequences of ignoring therapist effects in trials with longitudinal data: A simulation study. Journal of consulting and clinical psychology. 2018;86(9):711–25.

16. Svanborg P, Åsberg M. A new self-rating scale for depression and anxiety states based on the Comprehensive Psychopathological Rating Scale. Acta psychiatrica Scandinavica. 1994;89(1):21–8.

17. Kroenke K, Spitzer RL, Williams JBW. The PHQ-9: Validity of a brief depression severity measure. Journal of General Internal Medicine. 2001 Sep;16(9):606–13.

18. FRESCO DM, COLES ME, HEIMBERG RG, LIEBOWITZ MR, HAMI S, STEIN MB, et al. The Liebowitz Social Anxiety Scale: a comparison of the psychometric properties of self-report and clinician-administered formats. Psychological medicine. 2001 Aug;31(6):1025–35.

19. Antony MM, Coons MJ, McCabe RE, Ashbaugh A, Swinson RP. Psychometric properties of the social phobia inventory: Further evaluation. Behaviour research and therapy. 2006;44(8):1177–85.

20. Houck PR, Spiegel DA, Shear MK, Rucci P. Reliability of the self-report version of the panic disorder severity scale. Depression and anxiety. 2002;15(4):183–5.

21. Devlin NJ, Brooks R. EQ-5D and the EuroQol Group: Past, Present and Future. Appl Health Econ Health Policy. 2017 Apr;15(2):127-137. doi: 10.1007/s40258-017-0310-5. PMID: 28194657; PMCID: PMC5343080.

22. Saunders JB, Aasland OG, Babor TF, De La Fuente JR, Grant M. Development of the Alcohol Use Disorders Identification Test (AUDIT): WHO Collaborative Project on Early Detection of Persons with Harmful Alcohol Consumption-II. Addiction (Abingdon, England). 1993 Jun;88(6):791–804.

23. Berman AH, Bergman H, Palmstierna T, Schlyter F. Evaluation of the Drug Use Disorders Identification Test (DUDIT) in Criminal Justice and Detoxification Settings and in a Swedish Population Sample. European addiction research. 2005;11(1):22–31.

24. Bastien CH, Vallieres A, Morin CM. Validation of the insomnia severity index as an outcome measure for insomnia research. Sleep Med 2001; 2: 297-307.

25. Kessler RC, Adler L, Ames M, Demler O, Faraone S, Hiripi E, Howes MJ, Jin R, Secnik K, Spencer T, Ustun TB, Walters EE. The World Health Organization Adult ADHD Self-Report Scale (ASRS): a short screening scale for use in the general population. Psychol Med. 2005 Feb;35(2):245-56. doi: 10.1017/s0033291704002892. PMID: 15841682.

27. Ustün TB, Chatterji S, Kostanjsek N, et al. Developing the World Health Organization Disability Assessment Schedule 2.0. Bull World Health Organ. 2010; 88:815–823.

28. Sheehan DV, Lecrubier Y, Harnett-Sheehan K, Amorim P, Janavs J, Weiller E, Hergueta T, Baker R, Dunbar G: The Mini International Neuropsychiatric Interview (M.I.N.I.): The Development and Validation of a Structured Diagnostic Psychiatric Interview. J. Clin Psychiatry, 1998;59(suppl 20): 22-33.

29. Busner J, Targum SD. The clinical global impressions scale: applying a research tool in clinical practice. Psychiatry (Edgmont). 2007 Jul;4(7):28-37. PMID: 20526405; PMCID: PMC2880930.

30. Attkisson CC, Zwick R. The client satisfaction questionnaire. Evaluation and program planning. 1982;5(3):233–7.

31. Forsell E. Adaptive treatment strategies in internet-delivered cognitive behavior therapy: predicting and avoiding treatment failures. [Stockholm]: Karolinska Institutet; 2020.

32. Brooke J. System usability scale (SUS): a quick-and-dirty method of system evaluation user information. Reading, UK: Digital Equipment Co Ltd, 1986, 43.
